# Targeted literature review on use of tumor mutational burden status and programmed cell death ligand 1 expression to predict outcomes of checkpoint inhibitor treatment

**DOI:** 10.1186/s13000-020-0927-9

**Published:** 2020-01-30

**Authors:** Tina Krieger, Isobel Pearson, Judith Bell, Jim Doherty, Paul Robbins

**Affiliations:** 1 RTI Health Solutions, The Pavilion, Towers Business Park, Wilmslow Road, Didsbury, Manchester, M20 2LS UK; 2 Pfizer, Inc., La Jolla, USA

**Keywords:** Precision medicine, Biomarkers, Tumor mutational burden, Programmed cell death 1 receptor, Gene expression profiling

## Abstract

**Background:**

To achieve optimal outcomes, an individual approach is needed in the treatment and care of patients. The potential value of tumor mutational burden (TMB) status and/or programmed cell death ligand 1 (PD-L1) expression as biomarkers to predict which patients are most likely to respond to checkpoint inhibitors has been explored in many studies. The goal of this targeted literature review is to identify data available for TMB status and/or PD-L1 expression that predict response to checkpoint inhibitors and/or anti–cytotoxic T-lymphocyte–associated protein 4 (CTLA-4) antibodies.

**Methods:**

Targeted literature searches were performed using electronic medical databases (MEDLINE, Embase, and BIOSIS) and internet searches of specified sites. Bibliographies of key systematic literature reviews and meta-analyses also were reviewed for studies of interest.

**Results:**

The review identified 27 studies of non-small cell lung cancer (NSCLC), 40 studies of melanoma, 10 studies of urothelial cancer, and 5 studies of renal cell cancer indications. Studies also were identified in other cancer types, e.g., colorectal, breast, gastric, and Merkel cell cancer and squamous-cell carcinoma of the head and neck.

Twelve trials, including six in NSCLC and four in melanoma, evaluated TMB as a predictor of outcomes. A TMB of ≥10 mutations per megabase was shown to be an effective biomarker in the CheckMate 227 study. PD-L1 expression was included in the majority of identified studies and was found to predict response in in melanoma and in all types of NSCLC. Prediction of response was not a prespecified analysis in some studies; others had small sample sizes and wide confidence intervals. A clear predictive trend for PD-L1 expression was not identified in renal, breast, gastric, or Merkel cell cancer.

**Conclusion:**

Based on data contained in this review, assessment of TMB status and PD-L1 expression may help enhance the prediction of response to checkpoint inhibition in some tumors, such as NSCLC and melanoma. In this rapidly growing area of research, further exploratory biomarkers are being investigated including tumor-infiltrating lymphocytes, immune profiling (e.g., effector T cells or regulatory T cells), epigenetic signatures, T-cell receptor repertoire, proteomics, microbiome, and metabolomics.

## Background

### Precision Medicine and the Current Approach

According to the Precision Medicine Initiative, precision medicine is “an emerging approach for disease treatment and prevention that takes into account individual variability in genes, environment, and lifestyle for each person” [1]. Using this instead of a one-size-fits-all approach allows physicians to predict more accurately which treatment and prevention strategy will work for which patient groups for a specific disease. Despite sharing a histological phenotype, patients with the same cancer type may have distinct genetic elements, tumor microenvironments, and biochemical pathways and thus will require individualized treatment to optimize clinical benefit.

A current approach to precision medicine is to use genomic markers to target health care interventions. According to Phillips et al. [2], there were approximately 75,000 genetic tests on the market in 2017, of which about 86% were single-gene tests. The remaining tests included multi-analyte assays, noninvasive prenatal test, whole exome (protein coding only) sequencing (WES), and whole genome sequencing, so called next-generation sequencing.

The potential value of tumor mutational burden (TMB) and/or programmed cell death ligand 1 (PD-L1) expression biomarkers to enhance the prediction of which patients are most likely to respond to checkpoint inhibitors that target programmed cell-death protein or PD-L1 has been explored in multiple studies [3–6].

### TMB/Tumor Mutational Load

TMB, also known as tumor mutational load (TML), is a measure of the number of mutations within a tumor genome, sometimes defined as the total number of nonsynonymous point mutations per coding area of a tumor genome [7]. During their replication, tumor cells can develop multiple somatic mutations because of genetic instability that can alter protein-coding genes and potentially aberrant protein expression. These proteins are then broken down into peptides that may act as antigens or neoantigens once they are presented by the major histocompatibility complex on the tumor cell surface, which may be recognized by tumor-infiltrating lymphocytes (TILs), thereby triggering an immune response [8]. TMB has potential to predict the volume of neoantigens generated and thus the potential for eliciting an antitumor response and response to immunotherapy. Because of this predictive potential, TMB is emerging as a prominent independent biomarker for prediction of response to programmed cell death protein 1 (PD-1)/PD-L1 (PDx) pathway inhibitors in multiple cancer types [9], aided by the development of next-generation sequencing.

TMB as a biomarker was originally evaluated in advanced melanoma patients using WES data [10]. High mutational load showed a correlation in this study with a clinical benefit when treated with the cytotoxic T-lymphocyte–associated protein 4 (CTLA-4) inhibitor ipilimumab. The CheckMate 227 trial evaluated different nivolumab-based regimens versus chemotherapy in patients with non-small cell lung cancer (NSCLC) and showed that treatment with nivolumab plus ipilimumab in patients with a burden of at least 10 mutations per megabase was associated with longer progression-free survival (PFS) [4].

### Checkpoint Inhibitors PD-1/PD-L1 and CTLA-4

Naive T cells require both antigen presentation and a second costimulatory signal, usually CD28, to be activated. In contrast to CD28, PD-1 delivers a negative signal when bound to its ligands PD-L1 and programmed cell death ligand 2 (PD-L2) (Error! Reference source not found.). PD-1 suppresses T-cell activation by recruiting SHP-2, which inactivates ZAP-70, a crucial molecule in T-cell receptor signaling. PD-L1 can be produced in normal tissues and is crucial to prevent immune-mediated damage at the time of an inflammatory response as the activation of PD-1 inhibits T-cell effector functions [3, 11, 12]. Its expression is upregulated by interferon γ (IFN-γ) and other cytokines that are released by activated T cells.

Unfortunately, cells from many different human tumors can evade host immune surveillance by expressing PD-L1 on their surface. TILs recognize antigens expressed by tumor cells and presented by antigen-presenting cells, subsequently releasing IFN-γ leading to expression of PD-L1. An overexpression of PD-LI can result in an adaptive immune resistance within the tumor environment [3]. PD-L1 expression can also be driven by constitutive signaling pathways that involve phosphatase and tensin homolog, anaplastic lymphoma kinase, and epidermal growth factor receptor (EGFR) mutations [13].

Checkpoint inhibitory therapy is a form of cancer treatment currently under research globally for which James P. Allison and Tasuku Honjo received the Nobel Prize in Physiology or Medicine in 2018. The therapy inhibits immune checkpoints such as PD-L1 or PD-L2, PD-1, or CTLA-4, the first immune checkpoint receptor to be characterized. CTLA-4 is upregulated after T-cell activation and serves to down-regulate T-cell function to maintain T-cell homeostasis by binding to CD80; the same ligand is binding to CD28 for T-cell activation. Blocking CTLA-4 as the negative regulator of immune response can enhance antitumor immunity.

Currently, one antibody targeting CTLA-4 is clinically approved, ipilimumab, although others are in development. Nivolumab, cemiplimab, and pembrolizumab are the currently approved anti–PD-1 treatments for various cancer types. Approved anti–PD-L1 drugs that are on the market are atezolizumab, avelumab, and durvalumab. The activity of these checkpoint inhibitors has been studied in several cancers, including lung, breast, gastric, pancreatic, ovarian, renal cell, melanoma, and glioblastoma [14].

These immunotherapies may overcome immune inhibition and enhance or preserve the immune response against cancer cells. However, not all patients respond to anti–PD-1 or PD-L1 therapies, and so it is important to identify biomarkers that can predict clinical response [3].

The US Food and Drug Administration has approved four immunohistochemistry (IHC)-based assays to detect PD-L1 expression using diagnostic monoclonal antibodies: IHC 22C3, 28–8, SP142, and SP263 [15]. A review by Lantuejoul et al. [16] noted that a number of studies have shown a close analytical agreement for the Dako 22C3, Dako 28–8, and Ventana SP263 assays for tumor cell staining in NSCLC, with poor concordance for the Ventana SP142 assay and for immune cells.

This review will show the data available on TMB status and PD-L1 expression that predict response to PDx checkpoint inhibitors and anti-CTLA-4 antibodies.

## Methods

### Search Strategy

We searched MEDLINE, MEDLINE In-Process, Embase, and BIOSIS from August 2007 to April 2018. Search terms used combinations of free text and Medical Subject Heading (MeSH) terms. We used terms relating to TMB, PD-L1, PD-1, CTLA-4, precision medicine, cancer, and drugs of interest. No language or geographical limitations were applied.

To identify more recent studies, we also searched abstracts of the meetings for the American Association for Cancer Research, the Molecular Medicine Tri-Conference, and the European Society for Medical Oncology from 2015 through 2018 and the Society for Immunotherapy of Cancer and the American Society of Clinical Oncology from 2016 through 2018. In addition, we manually searched the reference lists of relevant systematic reviews and meta-analyses published in the 2 years prior to the search date for further studies of interest.

### Study Selection

We included studies of adults with any tumor type, treated with ipilimumab, tremelimumab, nivolumab, pembrolizumab, atezolizumab, avelumab, or durvalumab, that investigated biomarkers such as TMB status or the expression of checkpoint inhibitors PD-L1, PD-1, and CTLA-4 and were looking for subpopulations comparing different cutoffs. We included randomized, nonrandomized, and observational studies that reported at least one outcome of the following: overall survival (OS), PFS, time to progression, overall response rates, response rates, or relapse-free survival. We excluded studies in children, studies that did not have a treatment or outcome of interest, those that had a sample size of less than 50, and articles published before 2007 or abstracts before 2015.

Identified titles and abstracts were reviewed for inclusion against the predefined criteria by one researcher. To check for potential error or bias, a random 10% selection was also reviewed by another researcher; any differences were then resolved by consensus. Full-text articles were then obtained and reviewed in the same manner. Data of interest were extracted from the included studies by one reviewer and was verified by a second researcher. Extracted data included important study and baseline characteristics, OS, PFS, and response rates.

## Results

**Fig. 1:**
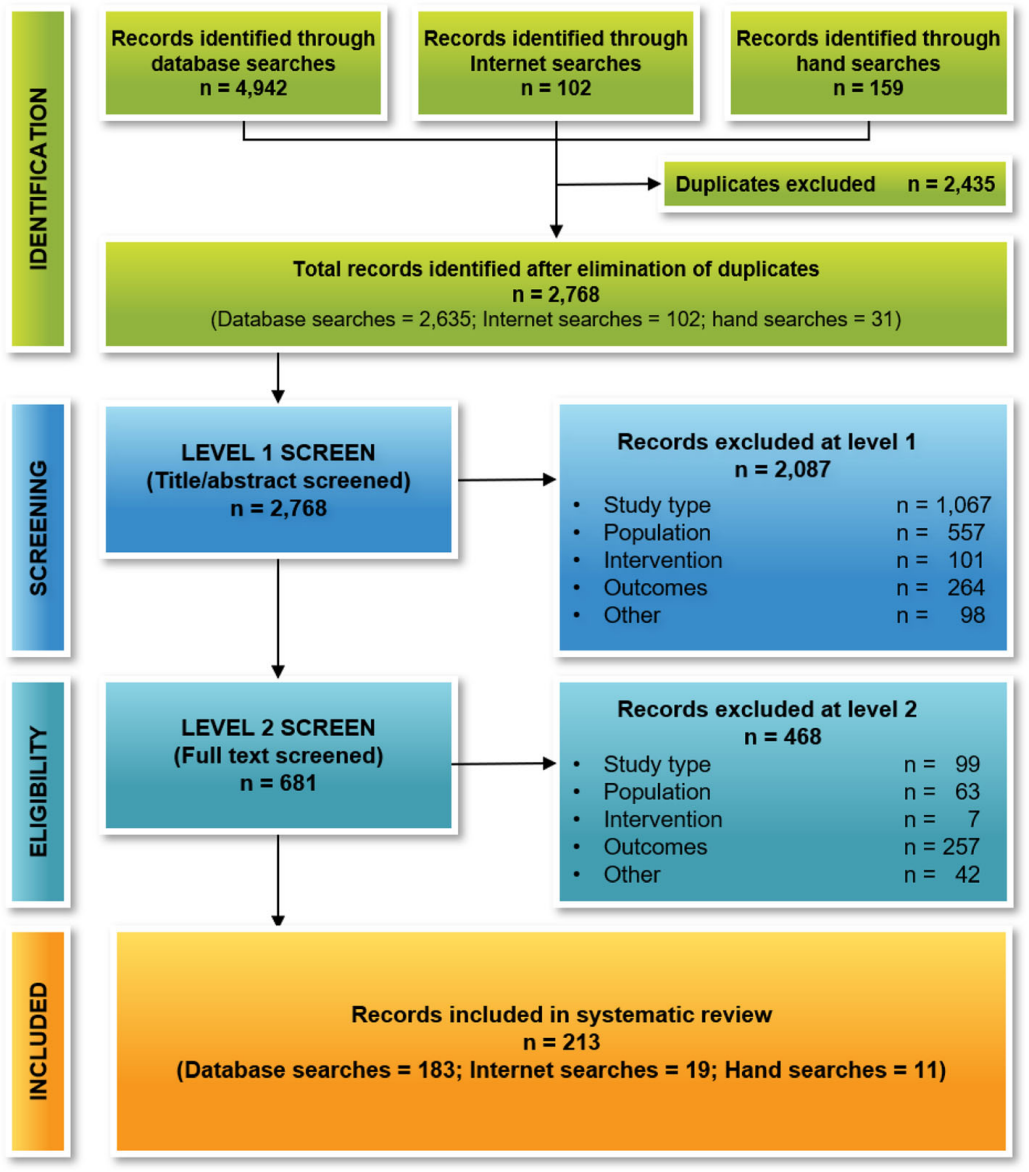
Preferred Reporting Items for Systematic Reviews and Meta-Analyses Diagram

The total number of articles identified and the screening process are shown in Fig. 1. After the elimination of duplicates, we reviewed 2768 titles and abstracts for inclusion and selected 681 articles to review at full text. After completion of screening process, we included 213 articles in this review: 100 primary studies and 113 secondary articles.

Of the 102 studies, we identified 27 studies for NSCLC, 40 studies for melanoma, 10 studies for urothelial cancer, and 5 studies for renal cell cancer indications. Fewer studies were identified for other cancer types, such as squamous-cell carcinoma of the head and neck (*n* = 2), colorectal cancer (*n* = 3), gastric cancer (*n* = 2), breast cancer (*n* = 1), Hodgkin’s disease (*n* = 1), Merkel cell cancer (*n* = 1), small cell lung cancer (n = 1), and pancreatic cancer (*n* = 1). Some studies presented data for multiple indications. Table 1 lists the indications we identified for each drug of interest. The studies were highly heterogenous, investigating a range of biomarker cutoffs with a variety of biomarker assays. The study characteristics and reported outcomes of all the studies of interest are presented in Additional file 1: Table S1.

**Table 1:** Indications Included in Identified Studies by Intervention

Drug	Indication(s)
CTLA-4 inhibitors	
Ipilimumab	Melanoma (*n* = 25), NSCLC (*n* = 3), mCRC (*n* = 1), SCLC (*n* = 1), RCC (*n* = 1), pancreatic (*n* = 1)
Tremelimumab	Melanoma (*n* = 3)
PD-1 inhibitors	
Nivolumab	NSCLC (n = 14), melanoma (*n* = 14), RCC (*n* = 3), urothelial (*n* = 2), SCCHN (*n* = 1), GC (n = 1), mCRC (*n* = 1), Hodgkin’s disease (*n* = 1), SCLC (*n* = 1)
Pembrolizumab	Melanoma (*n* = 9), NSCLC (*n* = 6), urothelial (*n* = 2), mCRC (*n* = 2), GC (*n* = 1), breast cancer (*n* = 1), SCCHN (*n* = 1)
PD-L1 inhibitors	
Atezolizumab	NSCLC (*n* = 6), melanoma (*n* = 2), urothelial (*n* = 3), RCC (*n* = 2)
Avelumab	NSCLC (*n* = 1), Merkel cell cancer (*n* = 1), urothelial (*n* = 1)
Durvalumab	NSCLC (*n* = 2), urothelial (*n* = 1)

*CTLA-4* cytotoxic T-lymphocyte-associated protein 4; *GC* gastric cancer; *mCRC* metastatic colorectal cancer; *NSCLC* non-small cell lung cancer; *PD-1* programmed cell death protein 1; *PD-L1* programmed death ligand 1; *RCC* renal cell cancer; *SCCHN* squamous-cell carcinoma of the head and neck; *SCLC* small cell lung cancer

### NSCLC

We identified 27 studies (69 references, including 3 pooled analyses) that presented outcome data of interest for NSCLC. Eleven studies presented data for nivolumab as treatment, 5 for atezolizumab, and 3 for pembrolizumab; the remaining studies reported data on other treatments or mixed treatments.

Six studies reported OS or PFS data for populations using TMB as a biomarker, as shown in Table 2. The cutoff points used included < 10, ≥ 10, < 12, ≥ 12, ≥ 13, < 14, ≥ 14, ≥ 16, < 16, < 20, and ≥ 20 mutations per megabase; some studies also reported TMB as low, medium, or high. Due to the varying definitions of TMB, it is difficult to draw direct comparisons between studies.

**Table 2:** Tumor Mutation Burden as Predictor of Non-small Cell Lung Cancer Outcome: OS and PFS Data

Trial/Author (Year)	Subpopulation or Population	Treatment	No. of Patients	OS		PFS	
				Median (95% CI), Months	HR(95% CI)	Median (95% CI), Months	HR(95% CI)
CheckMate 026 Carbone et al. (2017) [17] Socinski et al. (2016) [18]	High TMB	NIVO 3 mg/kg Q2W	47	18.3 (11.4-NE)	1.1 (0.64–1.88)	9.7 (5.1-NE)	0.62 (0.38–1.0)
		Platinum-based chemotherapy Q3W	60	18.8 (11.3-NE)		5.8 (4.2–8.5)	
	Low or medium TML	NIVO 3 mg/kg Q2W	111	12.7 (9.9–16.1)	0.99 (0.71–1.4)	4.1 (2.8–5.4)	1.82 (1.3–2.55)
		Platinum-based chemotherapy Q3W	94	13.2 (9.5–15.2)		6.9 (5.5–8.6)	
CheckMate 227 Hellmann et al. (2018) [4]	TMB ≥ 10 mutations per mb	NIVO + IPI	139	NR	NR	7.2 (5.5–13.2)	0.58 (97.5% CI, 0.41–0.81)
		Chemotherapy	160	NR	NR	5.5 (4.4–5.8)	
	TMB < 10 mutations per mb	NIVO + IPI	191	NR	NR	3.2 (2.7–4.3)	1.07 (0.84–1.35)
		Chemotherapy	189	NR	NR	5.5 (4.3–5.6)	
OAKa Rittmeyer et al. (2017) [6] Gadgeel et al. (2017) [19] Barlesi et al. (2016) [20] Hida et al. (2018) [21] Gandara et al. (2017) [22]	TMB ≥ 10	ATEZO vs. DTX	251	NR	0.69 (NR)	NR	0.73 (NR)
	TMB ≥ 16		158	NR	0.64 (NR)	NR	0.65 (NR)
	TMB ≥ 20		105	NR	0.65 (NR)	NR	0.61 (NR)
POPLARa Fehrenbacher et al. (2016) [23] Smith et al. (2016) [24] Mazieres et al. (2016) [25] Vansteenkiste et al. (2015) [26] Spira et al. (2015) [27] Gandara et al. (2017) [22]	TMB ≥ 10	ATEZO vs. DTX	96	NR	0.59 (NR)	NR	0.68 (NR)
	TMB ≥ 16		63	NR	0.56 (NR)	NR	0.57 (NR)
	TMB ≥ 20		42	NR	0.51 (NR)	NR	0.58 (NR)
Yaghmour (2016) [28]	TML: top quintile	≥ First line, NIVO or IPI	50 (overall patients)	NR	3.29 (0.75–25.53)	NR	NR
	TML: other quintiles			NR		NR	NR
B-F1RST Velcheti (2018) [29]	Blood-based TMB ≥ 12	ATEZO	22	NR	NR	3	0.95 (90% CI, 0.55–1.63)
	Blood-based TMB < 12		36	NR	NR	3.2	
	Blood-based TMB ≥ 14		14	NR	NR	3.4	0.73 (90% CI, 0.39–1.39)
	Blood-based TMB < 14		44	NR	NR	3.2	
	Blood-based TMB ≥ 16		11	NR	NR	9.5	0.49 (90% CI, 0.23–1.04)
	Blood-based TMB < 16		47	NR	NR	2.8	
	Blood-based TMB ≥ 20		8	NR	NR	9.5	0.23 (90% CI, 0.08–0.62)
	Blood-based TMB < 20		50	NR	NR	2.7	

*ATEZO* atezolizumab; *CI* confidence interval; *DTX* docetaxel; *HR* hazard ratio; *IPI* ipilimumab; *mb* megabase; *NE* could not be estimated/not reached; *NIVO* nivolumab; *NR* not reported; *OS* overall survival; *PFS* progression-free survival; *Q2W* every 2 weeks; Q3W every 3 weeks; *TMB* tumor mutational burden; *TML* tumor mutational load

^a^Blood based TMB

The most commonly applied TMB cutoff points were ≥ 10, ≥ 16, and ≥ 20 mutations per megabase. However, the studies that used these cutoff points used different definitions of TMB (blood or tissue based). B-F1RST [29] reported the greatest increase of median PFS (9.5 months) at the cutoff point ≥16 when using cutoff points ranging from ≥12 to ≥20.

The CheckMate 227 study [4] reported a median PFS of 3.2 and 7.2 months for TMB < 10 and TMB ≥ 10, respectively, for patients treated with first-line nivolumab 3 mg/kg plus ipilimumab 1 mg/kg. Nivolumab 3 mg/kg also was the first-line treatment used in CheckMate 026 [17]; the median PFS was 4.1 months for low or medium TMB and 9.7 for high TMB. A higher OS (18.3 vs. 12.7 months) was reported for the high-TMB group than for the low- or medium-TMB group. Interestingly, despite this study finding no association between PD-L1 expression and TMB, patients with both a high TMB and a PD-L1 expression of ≥50 had a higher response rate (75%) than patients with one (32–34%) or neither (16%) of these factors, suggesting that they are independent biomarkers predictive of response. It should be noted that the CheckMate 227 and CheckMate 026 studies used different methods to assess TMB (FoundationOne CDx assay and whole exome sequencing, respectively).

Two studies looked at TMB in second-line therapy and beyond when comparing atezolizumab and docetaxel therapy. Both OAK [6] and POPLAR [23] studies used the cutoff points ≥ 10, ≥ 16, and ≥ 20, and both reported an inverse relationship between TMB and OS HR. The OAK and POPLAR studies both used blood-based approaches to assess TMB. The OS HRs for the individual TMB cutoffs differed between studies: in OAK, they were 0.69, 0.64, and 0.65, respectively, for the three cutoff points, while in POPLAR, they were 0.59, 0.56, and 0.51, respectively [6, 23]. This difference could be attributed to the difference in population sizes or because patients with known EGFR or anaplastic lymphoma kinase mutations were excluded in Rittmeyer et al. [6]. In addition, increasing TMB may be prognostic but not predictive; i.e., tumors with higher levels of TMB would be less responsive to chemotherapy. As no confidence intervals (CIs) were reported for either study, it is not possible to determine the degree of significance of the difference in OS HR results between the studies.

Finally, Yaghmour et al. [28] investigated patients who had solid tumors, were treated with any checkpoint inhibitor, and had undergone next-generation sequencing. This study reported that OS was significantly higher in patients who were in the top quintile for TMB (hazard ratio [HR] = 5.78; 95% CI, 1.40–15.12). However, no significant difference was found in the population of patients who had NSCLC (*P* = 0.205; HR = undefined [95% CI, 0.53–25.70]).

Sixteen studies reported OS or PFS data in patients with NSCLC and with PD-L1 expression as a biomarker, as shown in Additional file 1: Table S2. The cutoff values for PD-L1 expression in tumor and/or immune cells used included < 1%, < 5%, < 10%, < 50, 1 to 49%, ≥ 1%, ≥ 5%, ≥ 10%, and ≥ 50%. Unfortunately, not all studies reported the PD-L1 expression cutoffs used. Additionally, study durations differed and, in some studies, the median OS or the upper limit of the CI was not reached.

The CheckMate 227 study [4] reported OS and PFS data in patients with NSCLC and both PD-L1 expression and TMB status as biomarkers (Additional file 1: Table S3).

The median OS for first-line treatment with nivolumab was highest in the subgroup with PD-L1 expression ≥50% [17]. In CheckMate 026 [17], the median OS for PD-L1 expression ≥1% was 13.7 months with nivolumab 3 mg/kg, compared with 20.2 months with nivolumab 10 mg/kg as treatment in CheckMate 012 [30]. The median OS for second-line treatment with nivolumab at a dose of 3 mg/kg ranged from 9.3 months to 17.7 months for patients with a PD-L1 expression ≥1%, 10.0 to 19.4 months for PD-L1 expression ≥5%, 11 to 19.9 months for PD-L1 expression ≥10%, and 8.7 to 10.5 months for PD-L1 expression < 1% in CheckMate 057 and 017 [31, 32].

For second-line treatment with atezolizumab (1200 mg), the median OS ranged from 15.5 to 15.7 months for PD-L1 expression ≥1%, 15.5 to 16.3 months for PD-L1 expression ≥5%, 15.1 to 20.5 months for ≥50%, and 9.7 to 12.6 months for PD-L1 expression < 1% in OAK and POPLAR [6, 23]. Rittmeyer et al. [6] differentiated the patient population into squamous and nonsquamous NSCLC. Comparing the median OS for PD-L1 expression ≥50% showed that survival appeared to be better in patients with nonsquamous NSCLC (22.5 months) than in patients with squamous NSCLC (17.5 months). Similar differences were shown for the other PD-L1 expression cutoffs.

### Melanoma

We identified 40 studies (53 references) that presented outcome data of interest for melanoma; however, limited OS and PFS data were available. Only 3 studies reported OS or PFS data using TMB as a biomarker (Table 3), while 5 studies reported OS or PFS data using PD-L1 expression (Additional file 1: Table S4).

**Table 3:** Tumor Mutation Burden as Predictor of Melanoma Outcome: OS and PFS Data

Trial/Author (Year)	Subpopulation or Population	Treatment	No. of Patients	OS		PFS	
				Median (95% CI), Months	HR (95% CI)	Median (95% CI), Months	HR(95% CI)
Johnson et al. (2016) [33]	High (> 23.1 mutations per mb)	NIVO, PEM, and ATEZO	65	NE	NR	NE	NR
	Intermediate (3.3–23.1 mutations per mb)		65	9.9 (NR)	NR	2.9 (NR)	NR
	Low (< 3.3 mutations per mb)		65	12.3 (NR)	NR	2.8 (NR)	NR
Roszik et al. (2016) [34]	Predicted TML ≤ 100	IPI	19	19.14 (NR)	0.35 (0.16–0.77)	NR	NR
	Predicted TML > 100		57	Undefined (NR)		NR	NR
Yaghmour et al. (2016) [28]	TML: top quintile	NIVO, PEM, and IPI	50 (overall patients)	NR	3.29(0.75–25.53)	NR	NR
	TML: other quintiles combined			NR		NR	NR

*ATEZO* atezolizumab; *CI* confidence interval; *HR* hazard ratio; *IPI* ipilimumab; *mb* megabase; *NE* could not be estimated/not reached; *NIVO* nivolumab; *NR* not reported; *OS* overall survival; *PFS* progression-free survival; *TML* tumor mutational load

Yaghmour et al. [28] reported that OS was higher in patients with a TMB in the top quintile (median genomic alterations = 16.5) than OS in patients with a TMB in the lower quintiles (median genomic alterations = 2) (HR = 3.29; 95% CI, 0.75–25.53). Patients were treated with nivolumab, pembrolizumab, or ipilimumab. Roszik et al. [34] also found that OS was higher in patients treated with ipilimumab who had a high predicted TMB (> 100) compared to those with a low predicted TMB (≤100) (median = undefined vs 582 days, *P* < 0.006). Additionally, Johnson et al. [33] and Yaghmour et al. [28] found that patients with a high TMB (> 23.1 mutations/megabase) had higher OS and PFS than those with intermediate TMB (3.3–23.1 mutations/megabase) or low TMB (< 3.3 mutations/megabase) (OS: median = not reached vs. 300 days vs. 375 days, *P* < 0.001; PFS: median = not reached vs. 89 days vs. 86 days, *P* < 0.001). Patients were treated with nivolumab, pembrolizumab, or atezolizumab.

Two studies (KEYNOTE-001 and KEYNOTE-006) used a PD-L1 expression cutoff of 1%, both investigating pembrolizumab [35–38]. The median OS was significantly higher in patients with PD-L1 expression ≥1% than in patients with PD-L1 expression < 1%, with an HR between 0.55 and 0.83 [35, 36]. Three other studies (CheckMate 066, CheckMate 067, CheckMate 069) used a PD-L1 expression cutoff of 5% [36, 39, 40], but the results are inconclusive.

### Other Indications

Limited literature was identified for other cancer types. We identified 10 studies of urothelial cancer, 5 studies of renal cell cancer, and 18 for other indications. Details of these studies are reported in Additional file 1: Table S1.

## Discussion

This review was conducted to identify published data regarding immune-related TMB status and PD-L1 expression that may predict response to PDx checkpoint inhibitors and anti-CTLA-4 antibodies. A total of 27 trials were identified for NSCLC, 40 trials for melanoma, 10 trials for urothelial cancer, and 5 trials for renal cell cancer; fewer trials were identified for the other cancer types. OS data were mainly identified for the NSCLC and melanoma indications. Data are available not only for different treatments but additionally for different lines of treatment.

A total of 12 studies reported on TMB, 6 of which were in NSCLC, where TMB appears to be a predictive biomarker for response. Five of the 6 studies reported PFS data. CheckMate 227 [4], OAK [6], POPLAR [23], and B-F1RST [29] showed an improved response at the cutoff points of ≥10 and ≥ 16 mutations per megabase, whereas CheckMate 026 [17] showed an improved response at a high TMB or a TMB in top quintiles, respectively. OS data also suggested that TMB could be an effective biomarker in NSCLC.

It should be noted that a number of studies questioning the potential benefit of the use of TMB assessment to predict response to checkpoint inhibitors in NSCLC have been published since the April 2018 cutoff for this review. Langer et al. [41] evaluated the relationship between TMB and outcomes in KEYNOTE-021 cohorts C (pembrolizumab plus carboplatin and pemetrexed) and G (randomized 1:1 to pembrolizumab plus carboplatin and pemetrexed or carboplatin and pemetrexed alone). In this study, TMB was not significantly associated with objective response rate, PFS, or OS for pembrolizumab plus chemotherapy or chemotherapy alone as first-line therapy for metastatic nonsquamous NSCLC. Garassino et al. [42] found that TMB was not significantly associated with efficacy of pembrolizumab plus chemotherapy or placebo plus chemotherapy as first-line therapy for metastatic nonsquamous NSCLC in KEYNOTE-189. Finally, TMB was not significantly associated with response to nivolumab plus ipilimumab compared with nivolumab for squamous NSCLC in Lung-MAP Sub-Study S1400I [43].

The IMvigor210 study [44, 45] in urothelial cancer and the Van Allen et al. [46] study in metastatic melanoma were able to show that TMB correlates with clinical benefit such as PFS and OS, but de Vlasco et al. [47] did not identify such a correlation in metastatic renal cell cancer when investigating a poor-risk group in the CheckMate 025 study.

Sixteen studies that reported OS or PFS data and PD-L1 expression as a biomarker were identified in patients with NSCLC. The studies reported the use of different assays and a number of different PD-L1 expression cutoffs, ranging from < 1% to ≥50%; not all of the studies reported the cutoff used. The median OS for first-line treatment with nivolumab was highest in the subgroup with PD-L1 expression ≥50% [17]. The second-line atezolizumab OAK study showed that PD-L1 expression ≥50% improved survival in nonsquamous NSCLC (22.5 months) compared with squamous NSCLC (17.5 months). Similar differences were shown for the other PD-L1 expression cutoffs. For NSCLC, PD-L1 expression data are reported in a number of studies, and PD-L1 expression appears to be an appropriate biomarker for predicting response for all NSCLC types.

In melanoma, 3 studies reported OS or PFS data and TMB. Roszik et al. [34] also found that OS was higher in patients treated with ipilimumab who had a high predicted TMB (> 100) compared to those with a low predicted TMB (≤100). Johnson et al. [33] and Yaghmour et al. [28] found that patients with a high TMB had higher OS and PFS than those with intermediate TMB or low TMB treated with nivolumab, pembrolizumab, or atezolizumab.

Additionally, 5 studies in melanoma reported OS or PFS data and the PD-L1 expression biomarker. The median OS was significantly higher in patients with PD-L1 expression ≥1% than in patients with PD-L1 expression < 1% in the pembrolizumab KEYNOTE-001 and KEYNOTE-006 trials, with an HR between 0.55 and 0.83 [35, 36].

### Data Gaps

The majority of data on TMB status and PD-L1 expression were identified for NSCLC, melanoma, and urothelial cancer but not the other cancer types. In addition, the majority of the biomarker data were identified for TMB and PD-L1 expression (biomarkers with limited data were PD-1 and CTLA-4 expression).

There is currently a lack of standardization on TMB calculation and reporting. The Friends of Cancer Research have an ongoing initiative to develop a consensus solution on how best to standardize current methods of TMB calculation [48]. Potentially useful biomarkers in squamous-cell NSCLC (Additional file 1: Table S1) are the SQ-cytoscore as used in Eberhardt et al. [49] or IFN-γ gene expression in both NSCLC types [50]. Iafolla and Juergens [51] suggest additional biomarkers that should be investigated further, including TILs, immunoprofiling (e.g., effector T cells or regulatory T cells), epigenetic signatures, T-cell receptor repertoire, proteomics, microbiome, and metabolomics. A few of these have been investigated in some of the studies presented in this review; however, the data are very limited and need further investigation.

Additional biomarkers such as high levels of microsatellite instability, which may relate to TMB with respect to the potential for increased antigenicity or tumor visibility to the immune system have been clinically validated and led to a multitumor approval for pembrolizumab.

While a clear predictive trend for PD-L1 expression was identified in studies of NSCLC and melanoma, this was not the case for other cancer types, including Merkel cell carcinoma, gastric, renal, and breast cancers. For these indications, other biomarkers are needed to enable individual prediction on whether a treatment will be successful for a patient or patient group.

The next generation of co-inhibitory receptor targets, such as lymphocyte-activation gene 3 and T-cell immunoglobulin and mucin-domain containing-3 [52], belong to the same receptor class as PD-1 and CTLA-4 but have unique functions, especially at tissue sites regulating distinct aspects of immunity, that are not yet fully understood. Herbst et al. [53] have evaluated these targets, but further investigation is needed.

With ongoing research into resistance to checkpoint inhibitors and also hyperprogressive disease [54, 55], there is potential for new biomarkers for response checkpoint inhibitors to be identified in the future.

## Conclusions

Based on the data contained in this review, assessment of TMB status and PD-L1 expression may help enhance the prediction of response to checkpoint inhibition in some tumors, such as NSCLC and melanoma. Carbone et al. [17] did a comparison which was not powered for statistical analysis that showed that in the nivolumab groups of the CheckMate 026 study, patients with both a high TMB and a PD-L1 expression level of 50% and above had a higher response rate compared to the other groups with a high TMB or a high PD-L1 expression. Following the date of publication cutoff for this review a number of studies have been published that question the potential benefit of the use of TMB assessment to predict response to checkpoint inhibitors in NSCLC and other tumors. Additional exploration into the significance of TMB across the range of tumor types is required to define the scope of its applicability as a biomarker.

Different cutoffs for TMB and percentage cutoffs for PD-L1 expression were used in the studies identified, and some studies did not report the cutoffs used. Additional biomarkers that should be investigated further include TILs, immunoprofiling (e.g., effector T cells or regulatory T cells), epigenetic signatures, T-cell receptor repertoire, proteomics, microbiome, and metabolomics.

## Supplementary information

**Additional file 1: Table S1.** Summary of Study Characteristics. **Table S2.** PD-L1: OS and PFS Data in NSCLC. **Table S3.** PD-L1 and TMB: OS and PFS Data in NSCLC. **Table S4.** PD-L1: OS and PFS Data in Melanoma

## Data Availability

All data generated or analyses during this study are included in this published article and its supplementary information files.

## Abbreviations

CI: Confidence interval
CTLA-4: Cytotoxic T-lymphocyte–associated protein 4
EGFR: Epidermal growth factor receptor
GC: Gastric cancer
HR: Hazard ratio
IHC: Immunohistochemistry
INF-γ: Interferon γ
mCRC: Metastatic colorectal cancer
MeSH: Medical Subject Heading
NA: Not applicable
NE: Not estimated
NSCLC: Non-small cell lung cancer
OS: Overall survival
PBO: Placebo
PD-1: Programmed cell death protein 1
PD-L1: Programmed cell death ligand 1
PD-L2: Programmed cell death ligand 2
PDx: Patient-derived xenograft
PFS: Progression-free survival
TIL: Tumor-infiltrating lymphocyte
TMB: Tumor mutational burden
TML: Tumor mutational load
WES: Whole exome (protein coding only) sequencing
