# Supplementary material for: Targeted literature review on use of tumor mutational burden status and programmed cell death ligand 1 expression to predict outcomes of checkpoint inhibitor treatment
Source: Diagn Pathol. 2020 Jan 30;15:6. doi: 10.1186/s13000-020-0927-9 (PMC6990470; doi:10.1186/s13000-020-0927-9)
Supplement: Supplementary file 1 — Additional file 1: Table S1. Summary of Study Characteristics. Table S2. PD-L1: OS and PFS Data in NSCLC. Table S3. PD-L1 and TMB: OS and PFS Data in NSCLC. Table S4. PD-L1: OS and PFS Data in Melanoma [file 13000_2020_927_MOESM1_ESM.docx]

Table S1. Summary of Study Characteristics

| Trial Acronym/ Author (Year) | Study Design/ Trial Phase | Treatment | Line of Therapy | Biomarker | Biomarker Cut-off Definition | Biomarker Assay Used | Outcomes Reported | | |
| --- | --- | --- | --- | --- | --- | --- | --- | --- | --- |
|  |  |  |  |  |  |  | OS | PFS | Response Rates |
| **NSCLC** | | | | | | | | | |
| BIRCH [S-[1-3](#_ENREF_1)] | Phase 2, single arm | ATEZO | 1, 2, or ≥ 3 | PD-L1 | ≥ 50%;1%-49% | SP142 IHC assay Ventana Medical Systems, Tucson, Arizona) | √ | √ | √ |
|  |  |  |  | *KRAS* | Mutant; wild type |  | √ | √ | √ |
|  |  |  |  | EGFR | Mutant; wild type |  |  |  |  |
| CheckMate 012 [S-[4](#_ENREF_4), [5](#_ENREF_5)] | Phase 1 study | NIVO | 1 | PD-L1 | ≥ 1%; < 1 % | NR | √ | √ | √ |
|  |  |  |  | EGFR | Mutant; wild type | NR | √ | √ | √ |
|  |  |  |  | *KRAS* | Mutant; wild type | NR | √ | √ | √ |
| CheckMate 017 [S-[6](#_ENREF_6)] | Phase 3 RCT | NIVO or DTX | 2 | PD-L1 | < 1%; ≥ 1%; < 5%; ≥ 5%; < 10%; ≥ 10% | IHC assay (Dako North America, Carpinteria, California) antihuman PD-L1 mAb (clone 28-8, Epitomics, Burlingame. California) | √ | √ | √ |
| CheckMate 017/057 [S-[7](#_ENREF_7), [8](#_ENREF_8)] | Follow-up of two phase 3 trials | NIVO or DTX | ≥ 1 | Cytoscore | High; low | Multivariate analyses of baseline serum cytokines | √ |  |  |
|  |  |  |  | PD-L1 | < 1%; ≥ 1%; ≥ 50% | NR | √ |  |  |
| CheckMate 026 [S-[9](#_ENREF_9), [10](#_ENREF_10)] | Phase 3 RCT | NIVO or platinum-based chemotherapy | 1 | PD-L1 | All: > 1%; ≥ 5%; ≥ 50% | Anti–PD-L1 antibody (28-8 antibody) | √ | √ | √ |
|  |  |  |  | TMB | High; low or medium | NR | √ | √ | √ |
|  |  |  |  | TMB and PD-L1 | High and PD-L1 ≥ 50%; high and PD-L1 1%-49%; low or medium and PD-L1 ≥ 50%; high and PD-L1 1%-49% |  |  |  | √ |
| CheckMate 057 [S-[11-13](#_ENREF_11)] | Phase 3 RCT | NIVO or DTX | ≥ 2 | PD-L1 | < 1%; ≥ 1%; < 5%  ≥ 5%; < 10%; ≥ 10% | IHC assay (Dako North America, Carpinteria, California) antihuman PD-L1 mAb (clone 28–8) | √ | √ | √ |
|  |  |  |  | EGFR | Positive; negative | NR | √ | √ |  |
|  |  |  |  | ALK | Translocation not detected | NR | √ | √ |  |
|  |  |  |  | *KRAS* | Positive | NR | √ | √ |  |
| CheckMate 063 [S-[14](#_ENREF_14)] | Phase 2 single arm | NIVO | ≥ 3 | PD-L1 | < 1%; ≥ 1%; < 5%  ≥ 5%;< 10%; 10% | IHC assay (Dako North America, Carpinteria, California) | √ | √ | √ |
|  |  |  |  | ALK translocation status | Negative; positive | NR | √ | √ |  |
| CheckMate 227 [S-[15](#_ENREF_15)] | Multipart phase 3 trial | NIVO + IPI, NIVO + platinum-based chemotherapy, NIVO monotherapy, or platinum-based chemotherapy alone | 1 | TMB^a^ | ≥ 10 mutations per mb  < 10 mutations per mb | Foundation One CDx assay (Foundation Medicine, Cambridge, Massachusetts) |  | √ | √ |
|  |  |  |  | TMB^a^ + PD-L1 | ≥ 10 mutations per mb + ≥ 1%  ≥ 10 mutations per mb + < 1%  ≥ 13 mutations per mb + ≥ 1% | FoundationOne CDx assay (Foundation Medicine, Cambridge, Massachusetts) |  | √ |  |
| CP1108 [S-[16-22](#_ENREF_16)] | A phase 1/2 escalation and dose-expansion study | DURVA | 1, 2, and ≥ 3 | PD-L1 and IFN-γ mRNA | None; all PD-L1 ≥ 25%; PD-L1 IHC < 25%; all IFN-γ+  IFN-γ+ and PD-L1 ≥ 25%; IFN-γ+ and PD-L1 < 25%; all IFN-γ–  IFN-γ– and PD-L1 ≥ 25%; IFN-γ– and PD-L1 < 25% | Analytically validated Ventana SP263 assay optimized for use on the automated BenchMark ULTRA platform (Ventana Medical Systems, Oro Valley, Arizona) and using Fluidigm Biomark for mRNA | √ |  | √ |
|  |  |  |  | PD-L1 | ≥ 25%; < 25% |  | √ | √ | √ |
| JAVELIN Solid Tumor [S-[23-32](#_ENREF_23)] | Dose-expansion cohort of an OL, phase 1 study | AVE | ≥ 2 | PD-L1 | < 1%; ≥ 1%; < 5%  ≥ 5%; < 10%; ≥ 10% | IHC assay (Dako North America, Carpinteria, California) anti–PD-L1 mAb clone 73–10 (Merck & Co. Inc., Kenilworth, New Jersey) | √ | √ | √ |
|  |  |  |  | EGFR | Wild type; mutant | NR | √ | √ |  |
|  |  |  |  | *KRAS* | Wild type; mutant | NR | √ | √ |  |
| KEYNOTE-001 [S-[33-40](#_ENREF_33)] | OL, phase 1, RCT | PEM | ≥ 1 | PD-L1 | ≥ 50%; 1%-49%; < 1%; positive; negative | IHC assay (early version of the PD-L1 22C3 IHC pharmDx assay; Dako North America, Carpinteria, California) | √ | √ | √ |
|  |  |  |  | *TP53* | *TP53* mutation | NR |  | √ |  |
|  |  |  |  | *KRAS* | Wild type; mutant |  |  | √ |  |
|  |  |  |  | EGFR | Wild type; mutant |  | √ |  |  |
|  |  |  |  | Baseline tumor size | Below median (10.2 cm); above median (10.2 cm) | NR |  |  | √ |
|  |  |  |  | *BRAF* | Wild type; mutant | NR | √ |  | √ |
| KEYNOTE-010 [S-[41-44](#_ENREF_41)] | Randomized, OL, phase 2/3 study | PEM or DTX | ≥ 1 | PD-L1 | ≥ 50%; 1%-49%; 1%‑24%; 25%-49%; 50%-74%; ≥ 75%; ≥ 1% | IHC assay with the murine 22C3 antihuman PD-L1 antibody | √ | √ | √ |
|  |  |  |  | EGFR | Mutant; wild type | NR | √ | √ |  |
| OAK [S-[45-49](#_ENREF_45)] | Randomized, OL, phase 3 study | ATEZO or DTX | > 1 | PD-L1 | ≥ 10%; ≥ 5%; ≥ 1%; < 1% | VENTANA SP142 PD-L1 IHC assay (Ventana Medical Systems, Tucson, AZ, USA) | √ | √ | √ |
|  |  |  |  | *KRAS* | Mutant; wild type | NR | √ |  |  |
|  |  |  |  | EGFR | Mutant; wild type | NR | √ |  |  |
|  |  |  | ≥ 2 | TMB | ≥ 10; ≥ 16; ≥ 20 | bTMB assay | √ |  |  |
| POPLAR [S-[49-54](#_ENREF_49)] | OL, phase 2 RCT | ATEZO or DTX | ≥ 2 | PD-L1 | ≥ 10%; ≥ 5%; ≥ 1%; < 1%; < median; ≥ median | VENTANA SP142 PD-L1 IHC assay (Ventana Medical Systems, Tucson, AZ, USA) | √ | √ | √ |
|  |  |  |  | PD-L2 | < Median; ≥ median | NR | √ |  |  |
|  |  |  |  | B7.1 | < Median; ≥ median | NR | √ |  |  |
|  |  |  |  | T effector- IFN-γ | < Median; ≥ median | NR | √ |  |  |
|  |  |  |  | TMB | ≥ 10; ≥ 16; ≥ 20 | NR | √ | √ |  |
| NCT00730639 Topalian et al. (2012) [S-[55](#_ENREF_55), [56](#_ENREF_56)] | Phase 1 dose-escalation, cohort-expansion study | NIVO | ≥ 2 | PD-L1 | ≥ 5%; < 5% | Murine antihuman PD-L1 monoclonal antibody, clone 5H1 | √ | √ | √ |
|  |  |  |  | EGFR | Mutant; wild type | NR |  |  | √ |
|  |  |  |  | *KRAS* | Mutant; wild type | NR |  |  | √ |
| NCT01375842 [S-[57](#_ENREF_57), [58](#_ENREF_58)] | Phase 1, single arm, dose escalation with expansion cohorts | ATEZO | NR | PD-L1 | < 1%; 1% to < 5%; 5% to < 10%; 1%-10%; ≥ 10%; ≥ 1% | SP142 assay (Ventana Medical Systems, Tucson, AZ, USA) |  | √ | √ |
|  |  |  |  | PD-L2 | Positive; positive and PD-L1 positive | SP142 assay (Ventana Medical Systems, Tucson, AZ, USA) |  |  | √ |
|  |  |  |  | ID01 | Positive | NR |  |  | √ |
|  |  |  |  | *LAG3* | Positive | NR |  |  | √ |
|  |  |  |  | TIM3 | Positive | NR |  |  | √ |
|  |  |  |  | CTLA-4 | Positive | NR |  |  | √ |
|  |  |  |  | B7-H3 | Positive | NR |  |  | √ |
|  |  |  |  | B7-H4 | Positive | NR |  |  | √ |
|  |  |  |  | ID01 | Positive and PD-L1+ | NR |  |  | √ |
|  |  |  |  | *LAG3* | Positive and PD-L1+ | NR |  |  | √ |
|  |  |  |  | TIM3 | Positive and PD-L1+ | NR |  |  | √ |
|  |  |  |  | CTLA-4 | Positive and PD-L1+ | NR |  |  | √ |
|  |  |  |  | B7-H3 | Positive and PD-L1+ | NR |  |  | √ |
|  |  |  |  | B7-H4 | Positive and PD-L1+ | NR |  |  | √ |
| Bagley et al. (2017) [S-[59](#_ENREF_59)] | Retrospective cohort study | NIVO | NR | Neutrophil-to-lymphocyte ratio | ≥ 5; < 5 | NR | √ | √ |  |
| De Castro et al. (2017) [S-[60](#_ENREF_60)] | Retrospective evaluation | Nonspecified anti–PD-1 and anti–PD-L1 | NR | LDH | Baseline ≥ 400; baseline < 400; < baseline at ≥ 3 evaluations; > baseline at ≥ 3 evaluations | NR | √ |  | √ |
| Gettinger (2015a) [S-[61](#_ENREF_61)] | Phase 1 study | NIVO | 1 | PD-L1 | ≥ 5%; < 5% | NR |  |  | √ |
| Kaderbhai et al. (2017) [S-[62](#_ENREF_62)] | Retrospective cohort study | NIVO | 2 and 3 | PD-L1 | ≥ 1%; < 1% | IHC using SP142 mAb | √ | √ | √ |
|  |  |  |  | EGFR | Mutant; wild type |  |  |  | √ |
|  |  |  |  | *KRAS* | Mutant; wild type |  |  |  | √ |
| Nomizo et al. (2017) [S-[63](#_ENREF_63)] | Retrospective medical record review | NIVO | ≥ 2 | PD-L1 SNPs | Different intron/ missense mutation | TaqMan® genotyping assay (Applied Biosystems, Foster City, California) analyzed with Applied Biosystems 7300 Real-Time PCR System |  | √ | √ |
| Pabla et al. (2017) [S-[64](#_ENREF_64)] | NR | ≥ 1 nonspecified checkpoint inhibitors | NR | PD-L1 | > 50% + inflamed; ≤ 50% + inflamed; ≤ 50% | PD-L1 (22C3) IHC and custom NGS cancer immune gene expression assay |  |  | √ |
| Roach et al. (2016) [S-[65](#_ENREF_65)] | Retrospective of phase 1 trial | PEM | NR | PD-L1 | ≥ 50% | PD-L1 IHC 22C3 pharmDx assay |  |  | √ |
| Sabari et al. (2017) [S-[66](#_ENREF_66)] | NR | NIVO, PEM, ATEZO, DURVA, IPI + NIVO | NR | PD-L1 | ≥ 50%; 0% | IHC |  |  | √ |
| Sorensen et al. (2016) [S-[67](#_ENREF_67)] | Retrospective study | PEM + platinum-based chemotherapy | 1 | PD-L1 | ≥ 50%; 1%-49%; < 1%; ≥ 1% | Prototype IHC assay with anti–PD-L1 22C3 antibody (Merck & Co., Inc., Kenilworth, New Jersey) | √ |  |  |
| Yaghmour et al. (2016) [S-[68](#_ENREF_68)] | Retrospective database search | NIVO, PEM, or IPI | ≥ 1 | Tumor mutational load | Top quintile; other quintiles combined | NGS cancer immune gene expression assay | √ |  |  |
| B‑F1RST [S-[69](#_ENREF_69)] | Phase 2 single-arm trial | ATEZO | 1 | Blood-based TMB | ≥ 12; < 12; ≥ 14; < 14; ≥ 16; < 16; ≥ 20; < 20 | NR |  | √ | √ |
| **SCLC** | | | | | | | | | |
| CheckMate 032 [S-[70](#_ENREF_70), [71](#_ENREF_71)] | Multicenter, phase 1/2 | NIVO and/or IPI | ≥ 2 | PD-L1 | < 1%; ≥ 1% | IHC assay (Dako North America, Carpinteria, California) antihuman PD-L1 mAb (clone 28–8) |  |  | √ |
| **Melanoma** | | | | | | | | | |
| CA184004 [S-[72](#_ENREF_72)] | Phase 2 trial | IPI | NR | Gene expression profiles | NR | NR | √ |  |  |
| CA209-004 [S-[73](#_ENREF_73)] | Phase 1 study | NIVO, IPI | Mixed | PD-L1 | ≥ 5%; < 5% | IHC assay (Dako North America, Carpinteria, California) antihuman PD-L1 mAb (clone 28–8) |  |  | √ |
|  |  |  |  | ALC | ALC < 1.0 × 10^3^ cells/L at weeks 5-7)  ALC ≥ 1.0 × 10^3^ cells/L at weeks 5-7) | NR |  |  | √ |
| CheckMate 037 [S-[74](#_ENREF_74)] | Phase 3 RCT | NIVO or chemotherapy | ≥ 2 | *BRAF* | Mutant; wild type | NR |  |  | √ |
|  |  |  |  | PD-L1 | Positive; negative | Automated Bristol-Myers Squibb/Dako IHC assay (Bristol-Myers Squibb, New York City, New York; Dako North America, Carpinteria, California) |  |  | √ |
| CheckMate 066 [S-[75](#_ENREF_75)] | Phase 3 RCT | NIVO or dacarbazine | 1 | PD-L1 | ≥ 5%; < 5% | IHC assay (Bristol-Myers Squibb, New York City, New York; Dako North America, Carpinteria, California) | √ |  | √ |
| CheckMate 066/067/069 [S-[76](#_ENREF_76)] | Phase 2 and phase 3 trials | NIVO +IPI or NIVO | NR | PD-L1 | ≥ 5%; < 5% | Dako IHC (Dako North America, Carpinteria, California) |  | √ | √ |
| CheckMate 067 [S-[77-79](#_ENREF_77)] | Phase 3 RCT | NIVO or IPI | 1 | PD-L1 | ≥ 5%; < 5% | IHC assay (Dako North America, Carpinteria, California) antihuman PD-L1 mAb (clone 28–8) |  | √ | √ |
|  |  |  |  | *BRAF* | Wild type; mutant | NR |  | √ |  |
| CheckMate 069 [S-[80](#_ENREF_80), [81](#_ENREF_81)] | Phase 1 dose-escalation study | NIVO and IPI or IPI and PBO | 1 | PD-L1 | ≥ 5%; < 5% | IHC assay (Bristol-Myers Squibb, New York City, New York; Dako North America, Carpinteria, California) |  |  | √ |
|  |  |  |  | *BRAF* | Wild type; mutant | NR |  | √ | √ |
| KEYNOTE-001 [S-[33-40](#_ENREF_33), [82](#_ENREF_82)] | OL, phase 1, RCT | PEM | 2 | PD-L1 | ≥ 1%; < 1% | IHC assay (PD-L1 IHC 22C3 pharmDx; Dako North America, Carpinteria, California) | √ | √ | √ |
| KEYNOTE-002 [S-[83](#_ENREF_83)] | Randomized, phase 2 trial | PEM or chemotherapy | ≥ 1 | *BRAF* | Mutant; wild type | NR |  | √ |  |
| KEYNOTE-006 [S-[84-86](#_ENREF_84)] | Randomized, OL, phase 3 trial | PEM or IPI | 1 and 2 | PD-L1 | Positive; negative | IHC analysis, 22C3 antibody (Merck & Co. Inc., Kenilworth, New Jersey) | √ | √ |  |
|  |  |  |  | *BRAF* | Wild type; mutant | NR | √ | √ |  |
| MDX010-020 [S-[87](#_ENREF_87)] | Randomized study | IPI and tremelimumab | ≥ 1 | CTLA-4 | *BRAF* (V600) or *NRAS* mutation; *BRAF*/*NRAS* wild type; *BRAF* and *NRAS* mutated | NR | √ |  |  |
| NCT00257205 Ribas et al. (2013) [S-[88](#_ENREF_88)] | Phase 3 RCT | Tremelimumab or SOC | 1 | LDH | ≤ ULN; 1-2 × ULN; > 2 × ULN | NR | √ |  |  |
|  |  |  |  | HLA | A2; Other | NR | √ |  |  |
| NCT00324155 Robert et al. (2011) [S-[89](#_ENREF_89)] | Randomized, DB, phase 3 study | IPI + dacarbazine or dacarbazine + PBO | 1 | LDH | > ULN; < ULN; > 2 × ULN; < 2 × ULN | NR | √ |  |  |
| NCT01176461 Weber et al. (2013) [S-[90](#_ENREF_90)] | Phase 1 study | NIVO | ≥ 2 | PD-L1 | ≥ 5%; < 5%; ≥ 1%; < 1% | IHC assay (Dako North America, Carpinteria, California) antihuman PD-L1 mAb (clone 28–8) |  |  | √ |
| Algazi et al. (2016) [S-[91](#_ENREF_91)] | Retrospective medical record review | PEM, NIVO, ATEZO | Multiple | LDH | Normal; elevated | NR | √ | √ |  |
| Arenberger et al. (2017) [S-[92](#_ENREF_92)] | Prospective single-arm study | IPI | Multiple | MAGE-3 | > 1 and ≤ 10; > 10 and ≤ 100; > 100 | Quantitative real-time RT-PCR: MIA, Melan-A/MART-1, MAGE-3, and gp100 (glycoprotein) | √ |  |  |
|  |  |  |  | Melan-A, gp100, MAGE-3, and melanoma inhibitory antigen | Increase in marker levels at visit 3 compared to visit 1  Significant mean reduction by more than 30% |  |  |  | √ |
| Chakravarti et al. (2017) [S-[93](#_ENREF_93)] | Prospective single-arm study | IPI | NR | Baseline ALC/U | NR | NR | √ | √ |  |
|  |  |  |  | Baseline LDH/100U | NR | NR | √ | √ |  |
|  |  |  |  | Tumor CTLA-4 percentage | < 20% (negative);  ≥ 20% (positive) | Antibody against CTLA-4, PD-1, and PD-L1 (Abcam, Cambridge, Massachusetts), FASL (Santa Cruz Biotechnologies, Santa Cruz, California), p-S6 and p-AKT (Cell Signaling, Danvers, Massachusetts), and BRAF-V600E (Ventana Medical Systems, Tucson, Arizona) | √ | √ |  |
|  |  |  |  | CTLA-4 intensity | 0; > 0 |  | √ |  |  |
|  |  |  |  | Tumor p-AKT intensity | ≤ 1 (negative); > 1 (positive) |  | √ |  |  |
|  |  |  |  | Tumor p-AKT/FAS-L intensity | –/–; +/–; +/+ |  | √ | √ |  |
|  |  |  |  | Tumor p-AKT/ CTLA-4 percentage | –/–; –/+; +/–; +/+ |  | √ | √ |  |
|  |  |  |  | Tumor p-AKT/p-S6 intensity | –/– |  |  | √ |  |
| Dick et al. (2016) [S-[94](#_ENREF_94)] | Retrospective database analysis | IPI | NR | LDH | Change in value after two cycles  Normal compared to elevated | NR | √ | √ |  |
| Diem et al. (2016) [S-[95](#_ENREF_95)] | Retrospective analysis | PEM or NIVO | NR | LDH | Normal; > ULN; > 10% CFB; ≤ 10% CFB | Serum LDH | √ |  | √ |
| Felix et al. (2016) [S-[96](#_ENREF_96)] | Prospective single-arm study | IPI | NR | LDH | < 500 U/L; ≥ 500 U/L | NR | √ |  |  |
|  |  |  |  | S100B | ≤ 0.15 µg/L; > 0.15 µg/L | S100 ELISA kit (DiaSorin, Stillwater, Minnesota) | √ |  |  |
|  |  |  |  | Anti-MICA | < 2; ≥ 2 | LABScreen assay | √ |  |  |
|  |  |  |  | Soluble MICA | ≤ 45 pg/mL; > 45 pg/mL | Human soluble MICA ELISA kit (Bamomab, Grafelfing, Germany) | √ |  |  |
|  |  |  |  | MIA | < 7.5 µg/L; ≥ 7.5 µg/L | MIA ELISA kit (Roche, Mannheim, Germany) | √ |  |  |
| Heppt et al. (2017) [S-[97](#_ENREF_97)] | Retrospective exploratory analysis | NIVO or IPI | NR | LDH | Normal; > ULN | NR | √ |  |  |
|  |  |  |  | CRP | Normal; > ULN | NR | √ |  |  |
|  |  |  |  | RLC | < 17.5%; ≥ 17.5% | NR | √ |  |  |
|  |  |  |  | REC | < 1.5%; ≥ 1.5% | NR | √ |  |  |
| Johnson et al. (2015) [S-[98](#_ENREF_98)] | Retrospective medical study | IPI | NR | PD-L1 | *NRAS* mutant; *BRAF* mutant; wild type | IHC assay (Dako North America, Carpinteria, California) antihuman PD-L1 mAb (clone 28-8) | √ | √ | √ |
| Johnson et al. (2016) [S-[99](#_ENREF_99)] | Retrospective medical study | NIVO, PEM, or ATEZO | ≥ 1 | TML | High (> 23.1 mutations per mb)  Intermediate (3.3-23.1 mutations per mb)  Low (< 3.3 mutations per mb) | Amendments-certified, hybrid capture-based NGS platform (FoundationOne, Foundation Medicine, Cambridge, Massachusetts) | √ | √ | √ |
| Ku et al. (2010) [S-[100](#_ENREF_100)] | Prospective, single-arm, compassionate use study | IPI | ≥ 2 | ALC | ≥ 1000/µL; < 1000/µL | NR | √ |  |  |
| Larkin et al. (2015b) [S-[101](#_ENREF_101)] | Pooled analysis of four clinical trials | NIVO | Multiple | *BRAF* | Wild type; Mutant | NR |  |  | √ |
|  |  |  |  | LDH | ≤ ULN and *BRAF* wild type; > ULN and *BRAF* wild type; ≤ ULN and *BRAF* mutation; > ULN and *BRAF* mutation | NR |  |  | √ |
|  |  |  |  | PD-L1 | ≥ 5% and *BRAF* mutation; < 5% and *BRAF* mutation; ≥ 5% and *BRAF* wild type; < 5% and *BRAF* wild type | NR |  |  | √ |
| Martens et al. (2016) [S-[102](#_ENREF_102)] | Cohort study | IPI | Multiple | LDH | ≤ 1.2; > 1.2; > 2.3 | NR | √ |  |  |
|  |  |  |  | RLC | < 10.5%; ≥ 10.5% | NR | √ |  |  |
|  |  |  |  | AMC | < 650/µL; ≥ 650/µL | NR | √ |  |  |
|  |  |  |  | AEC | < 50/µL; ≥ 50/µL | NR | √ |  |  |
|  |  |  |  | REC | < 1.5%; > 1.5% | NR | √ |  |  |
|  |  |  |  | CD4 + *CD25* + *FoxP3* + regulatory T cell | < 1.5%; ≥ 1.5% | NR | √ |  |  |
|  |  |  |  | *CD14*+ monocytes | < 28%; ≥ 28% | NR | √ |  |  |
|  |  |  |  | Lin-*CD14*+ HLA-DR/low MDSCs | < 5.1%; ≥ 5.1%; ≥ 9.5% | NR | √ |  |  |
| Morrison et al. (2017) [S-[103](#_ENREF_103)] | NR | One or more nonspecified checkpoint inhibitors | ≥ 1 | PD-L1 | PD-L1 positive + inflamed phenotype;  PD-L1 negative + inflamed phenotype;  PD-L1 negative | PD-L1 IHC and custom NGS immune gene expression assay |  |  | √ |
| Roh et al. (2017) [S-[104](#_ENREF_104)] | Longitudinal cohort study | IPI | NR | Mutational load/burden of copy number loss | High mutation + low copy loss;  High mutation + high copy loss;  Low mutation + low copy loss;  Low mutation + high copy loss | NR |  |  | √ |
| Roszik et al. (2016) [S-[105](#_ENREF_105)] | Retrospective samples | IPI or PEM | NR | Predicted TML | ≤ 100; > 100 | NR | √ | √ |  |
| Sade-Feldman et al. (2016) [S-[106](#_ENREF_106)] | Controlled clinical trial | IPI | ≥ 2 | MDSC | MDSCs > 55.5%; MDSCs < 55.5% | FACSCalibur using Cell Quest software (BD Biosciences, San Jose, California) | √ |  |  |
|  |  |  |  | LDH | LDH > 480 U/I; LDH < 480 U/I |  | √ |  |  |
|  |  |  |  | MDSC/LDH | High; MDSCs/LDH; low MDSCs/LDH |  | √ |  |  |
| Saenger et al. (2014) [S-[107](#_ENREF_107)] | Retrospective samples of phase 2 and phase 3 trials | Tremelimumab | ≥ 1 | Risk score | Low risk; intermediate risk; high risk | NR | √ |  |  |
| Sim and Elsheikh (2016) [S-[108](#_ENREF_108)] | Retrospective cohort study | NR | NR | PD-L1 | Tumor-infiltrating lymphocytes; melanoma tumor cells | NR |  | √ |  |
| Wilgenhof et al. (2013) [S-[109](#_ENREF_109)] | Prospective observational study | IPI | ≥ 2 | CRP | Baseline CRP ≤ 5 × ULN; Baseline CRP > 5 × ULN | NR | √ |  |  |
|  |  |  |  | ALC | ALC ≥ 800/mm^3^; ALC < 800/mm^3^; ALC at week 6 (2 doses);  800/mm^3^; ALC at week 6 (2 doses); < 800/mm^3^; ratio ALC ; 1  Ratio ALC ≤ 1 | NR | √ |  |  |
| Wistuba-Hamprecht et al. (2017) [S-[110](#_ENREF_110)] | Retrospective medical samples | IPI | NR | CD4 T cells | TCM ≤ 26.5%; TCM > 26.5%; TEM1 ≤ 17.5%; TEM1 > 17.5%; TEM3 ≤ 3.9%; TEM3 > 3.9%; TEM4 ≤ 0.2%; TEM4 > 0.2% | NR | √ |  |  |
|  |  |  |  | CD8 T cells | Naive ≤ 23.3%;  naive > 23.3%;  TCM ≤ 2.5%;  TCM > 2.5%;  TEM1 ≤ 13.0%; TEM1 > 13.0%; TEM3 ≤ 1.5%;  TEM3 > 1.5%;  TEM4 ≤ 1.9%;  TEM4 > 1.9%; TEMRA ≤ 23.8%; TEMRA > 23.8% | NR | √ |  | √ |
| Wu et al. (2017) [S-[111](#_ENREF_111)] | Retrospective medical samples | Various | ≥ 1 | ANGPT2 | Pretreatment:  > 3175 pg/mL; ≤ 3175 pg/mL; fold change:  ≥ 1.25 pg/mL; < 1.25 pg/mL | ANGPT2 plasma/serum samples measured using Magnetic Luminex Screening Assay kits (R&D Systems, Minneapolis, Minnesota) | √ |  | √ |
| Yaghmour et al. (2016) [S-[68](#_ENREF_68)] | Retrospective database search | NIVO, PEM, or IPI | ≥ 1 | TML | Top quintile; other quintiles combined | NGS reports generated from tissue biopsy specimens | √ |  |  |
| Yuan et al. (2014) [S-[112](#_ENREF_112)] | Retrospective medical samples | IPI | NR | VEGF | VEGF < 43 pg/mL; VEGF ≥ 43 pg/mL | MSD SECTOR Imager 2400 instrument (Meso Scale Discovery, Inc., Rockville, Maryland) | √ |  | √ |
| Zhou et al. (2017) [S-[113](#_ENREF_113)] | Retrospective medical samples | IPI + BEV or IPI or IPI + sargramostim or PEM | NR | Soluble PD-L1 | Soluble PD-L1 all < 1.4 ng/mL;  Soluble PD-L1 all ≥ 1.4 ng/mL;  Soluble PD-L1 < 0.5 ng/mL;  Soluble PD-L1 ≥ 0.5 ng/mL | NR |  |  | √ |
| Chasseuil et al. (2018) [S-[114](#_ENREF_114)] | Pilot monocentric retrospective study | NIVO | ≥ 1 | Leukocyte count | Univariate analysis;  multivariate analyses | NR | √ | √ | √ |
|  |  |  |  | Lymphocyte count |  |  |  |  |  |
|  |  |  |  | Leukocyte/ lymphocyte ratio |  |  |  |  |  |
|  |  |  |  | Neutrophil count |  |  |  |  |  |
|  |  |  |  | Neutrophil/ lymphocyte ratio |  |  |  |  |  |
|  |  |  |  | Monocyte count |  |  |  |  |  |
|  |  |  |  | Eosinophil count |  |  |  |  |  |
|  |  |  |  | Lactate dehydro-genase |  |  |  |  |  |
|  |  |  |  | C-reactive protein |  |  |  |  |  |
| Gaudy-Marqueste et al. (2017) [S-[115](#_ENREF_115)] | Retrospective cohort study | No targeted or immune therapy  *BRAF* ± MEK inhibitor alone  *BRAF* ± MEK inhibitor and immuno-therapy  Immuno-therapy alone  No immuno-therapy  Anti–PD-1 alone  IPI alone  IPI + anti–PD-1 | ≥ 1 | *BRAF* | Mutation; wild type | NR | √ |  |  |
| **Merkel cell carcinoma** | | | | | | | | | |
| JAVELIN Merkel 200 [S-[116](#_ENREF_116), [117](#_ENREF_117)] | Prospective, single-group, OL, phase 2 trial | AVE | ≥ 2 | PD-L1 | Positive; negative | Proprietary research-use-only assay (Dako North America, Carpinteria, California) based on anti–PD-L1 mAb (clone 73-10; Merck KGaA, Darmstadt, Germany) |  |  | √ |
|  |  |  |  | MCPyV | Positive; negative; not evaluable | mAb specific for Merkel cell polyomavirus large T antigen (Clone *CM2B4*; Santa Cruz Biotechnology, Dallas, Texas) |  |  | √ |
|  |  |  |  | PD-L1 and MCPyV | Positive/positive;  Positive/negative;  Negative/positive;  Negative/negative |  |  |  | √ |
| **Renal cell carcinoma** | | | | | | | | | |
| CheckMate 025 [S-[118](#_ENREF_118)] | Phase 3 RCT | NIVO or platinum-based chemotherapy | ≥ 2 | PD-L1 | ≥ 5%; < 5% | IHC assay (Dako North America, Carpinteria, California) antihuman PD-L1 mAb (clone 28–8) | √ |  |  |
| MDX-1106 [S-[87](#_ENREF_87)] | Blinded, randomized, phase 2 trial | NIVO | ≥ 1 | PD-L1 | < 5%; ≥ 5% | IHC assay (Dako North America, Carpinteria, California) antihuman PD-L1 mAb (clone 28–8) | √ | √ | √ |
| IMMotion-150 [S-[119](#_ENREF_119), [120](#_ENREF_120)] | Phase 2 RCT | ATEZO + BEV, ATEZO, or sunitinib | 1 | PD-L1 | Positive | IC SP142 IHC assay |  | √ | √ |
| NCT01375842 McDermott et al. (2016) [S-[121](#_ENREF_121)] | Phase 1, single arm, dose escalation | ATEZO | NR | PD-L1 | ≥ 1%; < 1% | SP142 assay | √ | √ | √ |
| CheckMate 214 [S-[122](#_ENREF_122)] | Phase 3 | NIVO + IPI or sunitinib | 1 | PD-L1 | < 1%; ≥ 1% | NR | √ | √ | √ |
| **Gastric or gastroesophageal cancer** | | | | | | | | | |
| ATTRACTION-02 [S-[123](#_ENREF_123)] | Phase 3 RCT | NIVO or PBO | ≥ 3 | PD-L1 | ≥ 1%; < 1% | 28-8 pharmDx assay | √ |  |  |
| KEYNOTE-059 [S-[124](#_ENREF_124)] | Global, multicohort, phase 2 study | PEM | 3 and 4 | PD-L1 | Positive; negative | IHC (22C3 antibody) |  |  | √ |
| **Colorectal cancer** | | | | | | | | | |
| CheckMate 142 [S-[125](#_ENREF_125), [126](#_ENREF_126)] | Phase 2 nonrandomized study | NIVO + IPI | ≥ 2 | PD-L1 | ≥ 1%; < 1% | Dako 28-8 pharmDx assay (Dako North America, Carpinteria, California) |  |  | √ |
|  |  |  |  | *BRAF* | Mutant | NR |  |  | √ |
|  |  |  |  | *KRAS* | Mutant |  |  |  | √ |
|  |  |  |  | *BRAF*/*KRAS* | Wild type |  |  |  | √ |
| KEYNOTE-164 [S-[127](#_ENREF_127), [128](#_ENREF_128)] | Global, multicenter, multicohort, phase 2 study | PEM | ≥ 3 | MSI-H | Positive tumors | IHC and/or PCR | √ | √ | √ |
| KEYNOTE-158 [S-[127](#_ENREF_127), [128](#_ENREF_128)] | Global, multicenter, multicohort, phase 2 study | PEM | ≥ 2 | MSI-H | Positive tumors | IHC and/or PCR | √ | √ | √ |
| **Urothelial cancer** | | | | | | | | | |
| CheckMate 032 [S-[129](#_ENREF_129), [130](#_ENREF_130)] | Phase 1/2 | NIVO | ≥ 2 | PD-L1 | ≥ 1%; < 1% | IHC assay (Dako North America, Carpinteria, California) antihuman PD-L1 mAb (clone 28–8) |  | √ | √ |
| CheckMate 275 [S-[131](#_ENREF_131), [132](#_ENREF_132)] | Single-arm, phase 2 study | NIVO | ≥ 2 | PD-L1 | < 1%; ≥ 1%; < 5%; ≥ 5% | Dako PD-L1 IHC 28-8 pharmDx kit (Dako North America, Carpinteria, California) | √ |  | √ |
| CP1108 [S-[133-136](#_ENREF_133)] | Phase 1/2 dose-escalation and dose‑expansion study | DURVA | Mixed | PD-L1 | ≥ 25%; < 25%; Low/negative | Ventana SP263 assay optimized for use on automated BenchMark ULTRA platform (Ventana Medical Systems, Tucson, Arizona) |  |  | √ |
|  |  |  |  | IFNGS | Positive: top tertile of IFNGS (*LAG3*, PD-L1, *CXCL9*, and IFN‑γ mRNAs)  Negative: not top tertile of IFNGS (*LAG3*, PD-L1, *CXCL9*, and IFN-γ mRNAs) | NR | √ | √ | √ |
| IMvigor-210 [S-[137-139](#_ENREF_137)] | Single-arm phase 2 study | ATEZO 1200 mg | 1  ≥ 2 | PD-L1 | IC 2/3 (≥ 5%); IC 1/2/3; IC 1 (≥ 1%, < 5%); IC 0 (< 1%) | VENTANA SP142 IHC assay (Ventana Medical Systems, Tucson, Arizona) | √ | √ | √ |
| IMvigor211 [S-[136](#_ENREF_136)] | Multicenter, OL, phase 3, randomized controlled trial | ATEZO or chemotherapy (physician's choice: vinflunine, paclitaxel, or DTX) | ≤ 3 | PD-L1 | IC 2/3 ≥ 5%; IC 1/2/3 ≥ 1% | VENTANA SP142 PD-L1 immunohistochemistry assay (Ventana Medical Systems, Tucson, Arizona) | √ | √ | √ |
|  |  |  |  | TMB | High (at or above median); low (less than median) | DNA extraction and preparation done with HistoGeneX NV (Antwerp, Belgium), DNA sequencing, genomic alteration detection, and FoundationOne test done by Foundation Medicine (Cambridge, Massachusetts) | √ |  |  |
|  |  |  |  | PD-L1 + TMB | IC 2/3 + high (at or above median);  IC 0/1 + high (at or above median) | VENTANA SP142 PD-L1 immunohistochemistry assay (Ventana Medical Systems, Tucson, Arizona)  DNA extraction and preparation done with HistoGeneX NV (Antwerp, Belgium) DNA sequencing, genomic alteration detection and FoundationOne test done by Foundation Medicine (Cambridge, Massachusetts) | √ |  |  |
| JAVELIN Solid Tumor [S-[140](#_ENREF_140)] | Dose-expansion cohort of a multicenter, OL, phase 1 study | AVE | ≥ 1 | PD-L1 | < 5%; ≥ 5% | Clone 73-10 |  |  | √ |
| KEYNOTE-045 [S-[141](#_ENREF_141), [142](#_ENREF_142)] | Randomized, OL, phase 3 trial | PEM or chemotherapy | 2 | PD-L1 | < 1%; ≥ 1%; < 10%; ≥ 10% | PD-L1 IHC 22C3 pharmDx assay (Dako North America, Carpinteria, California) | √ | √ | √ |
| KEYNOTE-052 [S-[143](#_ENREF_143), [144](#_ENREF_144)] | OL, multicenter, phase 2 study | PEM | 1 | PD-L1 | 18-gene expression profile and CPS; CPS ≥ 10%; CPS ≥ 1%; CPS ≥ 10% | NR |  |  | √ |
| NCT01375842 Petrylak et al. (2015) [S-[145](#_ENREF_145)] | Phase 1, single arm, dose escalation with expansion cohorts | ATEZO | NR | PD-L1 | IC 2/3; IC 0/1 | SP142 assay |  | √ | √ |
| CheckMate-032, IMvigor210, CA209-260 [S-[146](#_ENREF_146)] | Phase 2 randomized controlled trials | Anti–PD-1/ PD-L1 monotherapy | ≥ 1 | DNA damage response and repair | Deleterious; other; wild type | Memorial Sloan Kettering Integrated Molecular Profiling of Actionable Cancer Targets clinical sequencing assay | √ | √ |  |
| **SCCHN** | | | | | | | | | |
| CheckMate 141 [S-[147-150](#_ENREF_147)] | Phase 3, randomized controlled trial | NIVO or standard therapy | Mixed | PD-L1 | ≥ 1%; ≥ 5%; ≥ 10%; < 1%; < 5%; < 10% | IHC assay (Dako North America, Carpinteria, California) antihuman PD-L1 mAb (clone 28–8) | √ | √ | √ |
|  |  |  |  | p16 | Positive; negative | NR | √ |  | √ |
|  |  |  |  | p16 and PD-L1 | Positive and ≥ 1%; negative and ≥ 1%; positive and < 1%; negative and < 1% | NR | √ |  |  |
| KEYNOTE-012 [S-[151](#_ENREF_151), [152](#_ENREF_152)] | Phase 1b, multicenter, nonrandomized multicohort study | PEM | ≥ 1 | PD-L1 | ≥ 1%; < 1% | PD-L1 IHC 22C3 pharmDx assay (Dako North America, Carpinteria, California) 22C3 (Merck) anti–PD-L1 antibody | √ | √ | √ |
| **Classical Hodgkin's lymphoma** | | | | | | | | | |
| CheckMate 205 [S-[153](#_ENREF_153), [154](#_ENREF_154)] | Noncomparative, single-arm, phase 2 study | NIVO | ≥ 2 | 9p24·1 | Polysomy; copy gain; amplification | NR |  |  | √ |
|  |  |  |  | PD-L1 | Q1; Q2; Q3; Q4 | FISH with probes targeting PD-L1 (*CD274*), PD-L2 (*PDCD1LG2*) (both Empire Genomics, Williamsville, New York), and a centromeric region of chromosome 9 (*CEP 9*, control probe; Abbott Molecular, Des Plaines, Illinois) |  |  | √ |
| **Pancreatic** | | | | | | | | | |
| De Remigis et al. (2015) [S-[155](#_ENREF_155)] | Cohort study | GVAX or GVAX + IPI | NR | Thyroglobulin antibody seroconversion | Positive; negative | In-house ELISA and commercial ELISA QUANTA Lite and RIA KRONUS (Star, Idaho) thyroglobulin antibodies | √ |  |  |
| **Metastatic triple-negative breast cancer** | | | | | | | | | |
| KEYNOTE-086 [S-[156](#_ENREF_156)] | Phase 2, OL trial | PEM | ≥ 1 | PD-L1 | Positive and negative | NR | √ | √ | √ |
| **Multiple** | | | | | | | | | |
| ***Melanoma, renal cell carcinoma, and NSCLC*** | | | | | | | | | |
| NCT01375842 Herbst et al. (2014) [S-[57](#_ENREF_57), [58](#_ENREF_58), [121](#_ENREF_121), [145](#_ENREF_145)] | Phase 1, single arm, dose escalation with expansion cohorts | ATEZO | NR | PD-L1 | < 1%; 1% to < 5%; 5% to < 10%; ≥ 10%; increase in PD-L1 TC or IC of > 5% in patients with paired biopsies | SP142 assay |  | √ | √ |
| ***Metastatic tumors including breast, gastric, urothelial, and colorectal tumors*** | | | | | | | | | |
| Ayers et al. (2016) [S-[157](#_ENREF_157)] | Retrospective of KEYNOTE-012 and KEYNOTE-028 | PEM | NR | MSI-H | MSI-H; non-MSI-H | Microsatellite markers were analyzed by capillary electrophoresis |  |  | √ |
| ***SCLC, melanoma, and SCCHN*** | | | | | | | | | |
| Navarro et al. (2016) [S-[158](#_ENREF_158)] | Retrospective study | PEM, NIVO, or ATEZO | ≥ 1 | Immune gene signature | All signatures  T helper cells 1 high-tertile group  T helper cells 1 low-tertile group | RNA was analyzed using the NanoString PanCancer Immune Panel (NanoString Technologies, Seattle, Washington) |  | √ | √ |
| ***NSCLC, SCCHN, and melanoma*** | | | | | | | | | |
| Prat et al. (2017) [S-[159](#_ENREF_159)] | Retrospective of clinical trials | NIVO or PEM | ≥ 1 | Natural killer-cell expression | Low; high | Expression of 730 immune-related genes and 40 housekeeping genes using the nCounter platform (NanoString Technologies, Seattle, Washington) |  | √ |  |
| ***Any solid tumor with stage IV disease*** | | | | | | | | | |
| Yaghmour et al. (2016) [S-[68](#_ENREF_68)] | Retrospective database search | NIVO, PEM, or IPI | ≥ 1 | TML | Top quintile; other quintiles combined | NR | √ | √ | √ |
|  |  |  |  | PD-L1 | Positive; negative | IHC by the individual commercial labs | √ |  |  |
|  |  |  |  | PD-1 | Positive; negative |  | √ |  |  |
| ***NSCLC, mUC, or other advanced solid tumors*** | | | | | | | | | |
| FIR, BIRCH, POPLAR, OAK, IMVIGOR, PCD4989g [S-[160](#_ENREF_160)] | NR | ATEZO | ≥ 1 | Tissue TMB | ≥ 16 mutations per mb; < 16 mutations per mb | FoundationOne assay (Foundation Medicine, Cambridge, Massachusetts) |  |  | √ |

AEC = absolute eosinophil count; AKT = protein kinase B; ALC = absolute lymphocyte count; ALK = anaplastic lymphoma kinase; AMC = absolute monocyte count; ANGPT2 = angiopoietin-2 precursor; ATEZO = atezolizumab; AVE= avelumab; BEV= bevacizumab; bTMB = blood tumor mutational burden; CD25 = cluster of differentiation 25/interleukin 2 receptor alpha; CFB = change from baseline; CPS = combined positive score; CRP = C-reactive protein; CTLA-4 = cytotoxic T-lymphocyte–associated protein 4; *CXCL9* = chemokine (C-X-C) motif ligand 9; DB= double-blind; DNA = deoxyribonucleic acid; DTX = docetaxel; DURVA = durvalumab; EGFR = epidermal growth factor receptor; ELISA = enzyme-linked immunosorbent assay; FISH = fluorescence in situ hybridization; *FOXP3* = Forkhead Box P3; GVAX = pancreatic cancer vaccine; HLA = human leukocyte antigen; HLA-DR = human leukocyte antigen-D related; IC = immune cell; IFN-γ = interferon gamma; IFNGS = interferon gene signature; IHC = immunohistochemistry; IPI = ipilimumab; *KRAS* = KRAS Proto-Oncogene, GTPase; *LAG3* = lymphocyte-activation gene 3; LDH = lactate dehydrogenase; mAb = monoclonal antibody; MAGE-3 = melanoma-associated antigen 3; MART-1 = melanoma antigen recognized by T cells 1; mb= megabase; MCPyV = Merkel cell polyomavirus; MDSC = myeloid-derived suppressor cell; MEK = mitogen-activated protein kinase enzyme; MIA = multiplex immunoassay; MICA = major histocompatibility complex class I-related chain A; mRNA = messenger ribonucleic acid; MSI-H = high-level microsatellite instability; mUC = metastatic urothelial cancer; NGS = next-generation sequencing; NIVO= nivolumab; NR = not reported; NSCLC = non–small cell lung cancer; OL = open-label; OS = overall survival; p-AKT = phospho-AKT; p‑S6 = phospho-S6 ribosomal protein; PBO = placebo; PCR = polymerase chain reaction; PD-1 = programmed cell death protein 1; PD-L1 = programmed cell death ligand 1; PD-L2 = programmed cell death ligand 2; PEM= pembrolizumab; PFS = progression-free survival; Q = quarter; RCT= randomized controlled trial; REC = relative eosinophil count; RLC = relative lymphocyte count; RNA = ribonucleic acid; RT-PCR = reverse transcription polymerase chain reaction; SCCHN = squamous-cell carcinoma of the head and neck; SCLC = small cell lung cancer; SOC = standard of care; TC = tumor cell; TCM = central memory T cell; TEM1 = effector memory T cell-1; TEM3 = effector memory T cell-3; TEM4 = effector memory T cell-4; TEMRA = effector memory T cell RA; TIM3 = T‑cell immunoglobulin and mucin-domain containing-3; TMB = tumor mutational burden; TML = tumor mutational load; ULN = upper limit of normal; VEGF = vascular endothelial growth factor.

^a^ Defined as the number of somatic, coding base substitutions and short insertions and deletions per megabase of genome examined.

Table S2. PD-L1: OS and PFS Data in NSCLC

| Trial Acronym/ Author (Year) | Population | Treatment | No. of Patients | OS | | PFS | |
| --- | --- | --- | --- | --- | --- | --- | --- |
|  |  |  |  | Median (95% CI), Months | HR (95% CI) | Median (95% CI), Months | HR (95% CI) |
| BIRCH [S-[1-3](#_ENREF_1)] | PD-L1 ≥ 50% | First-line ATEZO | 65 | NE (12-NE) | NR | 5.6 (2.7-8.3) | NR |
|  |  | Second-line ATEZO | 122 | 15.1 (12-NE) | NR | 4 (1.5-5.5) | NR |
|  |  | ≥ Third ATEZO | 115 | 17.5 (11.1-NE) | NR | 4.1 (2.8-5.6) | NR |
|  | PD-L1 10-49% | First-line ATEZO | 74 | 20.1 (NE) | NR | 5.3 (2.8-6.9) | NR |
|  |  | Second-line ATEZO | 146 | 15.5 (11.9-NE) | NR | 2.6 (1.4-2.8) | NR |
|  |  | ≥ Third ATEZO | 136 | 11 (7.5-14.9) | NR | 2.7 (1.5-2.8) | NR |
|  | PD-L1 TC 3 or IC 3 | First-line ATEZO | 65 | 26.9 (12-NE) | NR | 7.3 (4.9-12) | NR |
|  | PD-L1 TC 2 or IC 2b |  | 73 | 23.5 (18.1-NE) | NR | 7.6 (4-9.7) | NR |
| CheckMate 012 [S-[4](#_ENREF_4), [5](#_ENREF_5)] | PD-L1 ≥ 1% | NIVO 10 mg/kg | 23 | 20.2 (NR) | NR | 6 (NR) | NR |
|  | PD-L1 < 1 % |  | 21 | 19.2 (NR) | NR | 5.2 (NR) | NR |
| CheckMate 017 [S-[6](#_ENREF_6)] | PD-L1 < 1% | NIVO 3 mg/kg | 54 | 8.7 (NR) | 0.58 (0.58-0.92) | 3.1 (NR) | 0.66 (0.43-1.0) |
|  |  | DTX 75 mg/mg | 52 | 5.9 (NR) |  | 3 (NR) |  |
|  | PD-L1 ≥ 1% | NIVO 3 mg/kg | 63 | 9.3 (NR) | 0.69 (0.45-1.1) | 3.3 (NR) | 0.67 (0.44-1.0) |
|  |  | DTX 75 mg/m^2^ | 56 | 7.2 (NR) |  | 2.8 (NR) |  |
|  | PD-L1 < 5% | NIVO 3 mg/kg | 75 | 8.5 (NR) | 0.7 (0.47-1.0) | 2.2 (NR) | 0.75 (0.52-1.1) |
|  |  | DTX 75 mg/m^2^ | 69 | 6.4 (NR) |  | 2.9 (NR) |  |
|  | PD-L1 ≥ 5% | NIVO 3 mg/kg | 42 | 10 (NR) | 0.53 (0.31-0.89) | 4.8 (NR) | 0.54 (0.32-0.9) |
|  |  | DTX 75 mg/m^2^ | 39 | 6.1 (NR) |  | 3.1 (NR) |  |
|  | PD-L1 < 10% | NIVO 3 mg/kg | 81 | 11 (NR) | 0.7 (0.48-1.0) | 2.3 (NR) | 0.7 (0.49-0.99) |
|  |  | DTX 75 mg/m^2^ | 75 | 6.1 (NR) |  | 2.8 (NR) |  |
|  | PD-L1 ≥ 10% | NIVO 3 mg/kg | 36 | 8.2 (NR) | 0.5 (0.28-0.89) | 3.7 (NR) | 0.58 (0.33-1.0) |
|  |  | DTX 75 mg/m^2^ | 33 | 7.1 (NR) |  | 3.3 (NR) |  |
| CheckMate 026 [S-[9](#_ENREF_9), [10](#_ENREF_10)] | PD-L1 > 1% | NIVO 3 mg/kg | 271 | 13.7 (11.8-15.4) | 1.07 (0.86-1.33) | 4.2 (3.1-5.5) | 1.17 (0.95-1.43) |
|  |  | Platinum-based CT | 270 | 13.8 (11-17) |  | 5.8 (5.4-6.9) |  |
|  | PD-L1 ≥ 5% | NIVO 3 mg/kg | 211 | 14.4 (11.7-17.4) | 1.02 (0.8-1.3) | 4.2 (3.0-5.6) | 1.15 (0.91-1.45) |
|  |  | Platinum-based CT | 212 | 13.2 (10.7-17.1) |  | 5.9 (5.4-6.9) |  |
|  | PD-L1 ≥ 50% | NIVO 3 mg/kg | 88 | 15.9 (NR) | 0.9 (0.63-1.29) | 5.4 (NR) | 1.07 (0.77-1.49) |
|  |  | Platinum-based CT | 126 | 13.9 (NR) |  | 5.8 (NR) |  |
| CheckMate 057 [S-[11-13](#_ENREF_11)] | PD-L1 < 1% | NIVO 3 mg/kg | 108 | 10.5 (NR) | 0.9 (0.66-1.24) | 2.1 | 1.19 (0.88-1.61) |
|  |  | DTX 75 mg/m^2^ | 101 | 10.1 (NR) |  | 3.6 |  |
|  | PD-L1 ≥ 1% | NIVO 3 mg/kg | 123 | 17.7 (NR) | 0.59 (0.43-0.82) | 4.2 | 0.7 (0.53-0.94) |
|  |  | DTX 75 mg/m^2^ | 123 | 9 (NR) |  | 4.5 |  |
|  | PD-L1 < 5% | NIVO 3 mg/kg | 136 | 9.8 (NR) | 1.01 (0.77-1.34) | 2.1 | 1.31 (1.01-1.71) |
|  |  | DTX 75 mg/m^2^ | 138 | 10.1 (NR) |  | 4.2 |  |
|  | PD-L1 ≥ 5% | NIVO 3 mg/kg | 95 | 19.4 (NR) | 0.43(0.30-0.63) | 5 | 0.54 (0.39-0.76) |
|  |  | DTX 75 mg/m^2^ | 86 | 8.1 (NR) |  | 3.8 |  |
|  | PD-L1 < 10% | NIVO 3 mg/kg | 145 | 9.9 (NR) | 1 (0.76-1.31) | 2.1 | 1.24 (0.96-1.61) |
|  |  | DTX 75 mg/m^2^ | 145 | 10.3 (NR) |  | 4.2 |  |
|  | PD-L1 ≥ 10% | NIVO 3 mg/kg | 86 | 19.9 (NR) | 0.4 (0.26-0.59) | 5 | 0.52 (0.37-0.75) |
|  |  | DTX 75 mg/m^2^ | 79 | 8 (NR) |  | 3.7 |  |
| CP1108 [S-[16-22](#_ENREF_16)] | PD-L1 TC ≥ 25% | Second line, DURVA | 46 | 17.8 (7.9-22.4) | NR | NR | NR |
|  | PD-L1 TC < 25% |  | 24 | 8.2 (4.9-15.5) | NR | NR | NR |
|  | PD-L1 TC ≥ 25% | ≥ Third line, DURVA | 59 | 13 (6-NE) | NR | NR | NR |
|  | PD-L1 TC < 25% |  | 82 | 7.1 (4.3-10) | NR | NR | NR |
|  | IFN-ɣ mRNA any and PD-L1 IHC+ (≥ 25%) | Mixed line, DURVA | 43 | NE (8.8-NE) | 0.4 | NR | NR |
|  | IFN-ɣ mRNA any and PD-L1 IHC– |  | 20 | NE (6.5-NE) | 0.38 | NR | NR |
|  | IFN-ɣ mRNA any and PD-L1 IHC+ (≥25%) |  | 42 | 9.7 (8.8-NE) | 0.64 | NR | NR |
|  | IFN-ɣ mRNA any and PD-L1 IHC– |  | 52 | 5.9 (4.1-10.2) | NR | NR | NR |
|  | PD-L1 (high): ≥ 25% of TC expressed PD‑L1 | First line, DURVA | 109 | 15.4 (9.7-22.4) | NR | NR | NR |
|  | PD-L1 (low/negative): < 25% of TC |  | 108 | 7.6 (5.6-10) | NR | NR | NR |
| JAVELIN Solid Tumor [S-[23-32](#_ENREF_23)] | PD-L1 ≥ 1% positive | ≥ Second line, AVE | 122 | 8.9 (8-NE) | 0.64 (0.34-1.2) | 2.8 (2.4-4.1) | 0.4 (0.27-0.75) |
|  | PD-L1 ≥ 1% negative |  | 20 | 4.6 (2.8-NE) |  | 1.4 (1.3-1.6) |  |
|  | PD-L1 ≥ 5% positive |  | 84 | 10.6 (7.9-NE) | 1.14 (0.7-1.02) | 2.7 (1.5-4.2) | NR |
|  | PD-L1 ≥ 5% negative |  | 58 | 8.4 (5.6-NE) |  | 1.8 (1.4-2.8) | NR |
|  | PD-L1 ≥ 25% positive |  | 53 | 8.44 (6-NE) | 1.14 (0.7-1.85) | 2.7 (1.5-4.2) | 0.79 (0.53-1.18) |
|  | PD-L1 ≥ 25% negative |  | 89 | 8.57 (7.16-NE) |  | 2.5 (1.4-3.2) |  |
|  | PD-L1 ≥ 10% positive |  | 27 | 8.5 (3.9-NE) | 1.2 (0.68-2.14) | 1.9 (1.3-3.5) | 1.19 (0.74-1.92) |
|  | PD-L1 ≥ 10% negative |  | 115 | 8.9 (7.9-NE) |  | 2.6 (1.5-3.5) | NR |
| KEYNOTE-001 [S-[33-40](#_ENREF_33)] | PD-L1:  PS ≥ 50% training group | ≥ First line, PEM  2 mg/kg Q3W or 10 mg/kg Q3W or 10 mg/kg Q2W | 38 | 13.7 (6.9-NE) | NR | 4.5 (1.9-12.5) | NR |
|  | PD-L1:  PS 1%-49% training group |  | 43 | 5.9 (4.2-8.2) | NR | 2.1 (2-2.9) | NR |
|  | PD-L1:  PS < 1% training group |  | 40 | 6.7 (3.9-10) | NR | 2.1 (1.8-2.5) | NR |
|  | PD-L1: PS ≥ 50% validation group |  | 73 | NE (NE-NE) | NR | 6.4 (4.2-NE) | NR |
|  | PD-L1:  PS 1%-49% validation group |  | 103 | 10.6 (7.3-NE) | NR | 4.1 (2.3-4.4) | NR |
|  | PD-L1: PS < 1% validation group |  | 28 | 10.4 (7.3-NE) | NR | 4 (2.1-6.2) | NR |
|  | PD-L1 positive: overall |  | 101 | 22.1 (17.1-27.2) | NR | 6.2 (4.1-8.6) | NR |
|  | PD-L1 positive: TPS ≥ 50% |  | 27 | NE (22.1-NE) | NR | 12.5 (6.2-NE) | NR |
|  | PD-L1 positive:  TPS 1%-49% |  | 52 | 19.5 (10.7-22.2) | NR | 4.2 (3.1-6.4) | NR |
|  | PD-L1 positive: TPS < 1% |  | 12 | 14.7 (3.4-NE) | NR | 3.5 (2.1-19) | NR |
|  | PD-L1: TPS ≥ 1% |  | 79 | 22.2 (16.7-31.5) | NR | NR | NR |
|  | PD-L1: TPS ≥ 50% |  | 27 | 34.9 (20.3-NE) | NR | NR | NR |
|  | PD-L1:  TPS 1%-49% |  | 52 | 19.5 (10.7-26.3) | NR | NR | NR |
|  | PD-L1: TPS ≥ 1% |  | 306 | 11.1 (8.3-14) | NR | NR | NR |
|  | PD-L1: TPS ≥ 50% |  | 138 | 15.4 (10.5-18.5) | NR | NR | NR |
|  | PD-L1:  TPS 1%-49% |  | 90 | 8.5 (6-12.7) | NR | NR | NR |
|  | PD-L1: TPS < 1% |  | 90 | 8.6 (5.5-10.6) | NR | NR | NR |
|  | PD-L1: TPS ≥ 1% |  | 79 | 22.1 (16.7-27.2) | NR | NR | NR |
|  | PD-L1: TPS ≥ 50% |  | 27 | NE (22.1-NE) | NR | NR | NR |
|  | PD-L1:  TPS 1%-49% |  | 52 | 19.5 (10.7-22.2) | NR | NR | NR |
|  | PD-L1: TPS < 1% |  | 12 | 14.7 (3.4-NE) | NR | NR | NR |
|  | PD-L1: TPS ≥ 1% |  | 306 | 11.3 (8.3-14) | NR | NR | NR |
|  | PD-L1: TPS ≥ 50% |  | 138 | 15.4 (10.6-18.5) | NR | NR | NR |
|  | PD-L1:  TPS 1%-49% |  | 168 | 8.2 (6-12.7) | NR | NR | NR |
|  | PD-L1: TPS < 1% |  | 90 | 8.6 (5.5-12) | NR | NR | NR |
|  | PD-L1 ≥ 50% |  | 17 | NE (NE-NE) | NR | NE (2.4-NE) | NR |
|  | PD-L1 1%-49% |  | 31 | NE (8.6-NE) | NR | 4.4 (3.6-6.4) | NR |
|  | PD-L1 < 1% |  | 7 | 7.3 (3.4-NE) | NR | 3.4 (2.1-4.2) | NR |
| KEYNOTE-010 [S-[41-44](#_ENREF_41)] | PD-L1: TPS ≥ 1% | > First line, PEM 2 mg/kg Q3W | 344 | 10.4 (9.4-11.9) | 0.71 (0.58-0.88) | 3.9 (3.1-4.1) | 0.88 (0.74-1.05) |
|  | PD-L1: TPS ≥ 1% | > First line, PEM 10 mg/kg Q3W | 346 | 12.7 (10-17.3) | 0.61 (0.49-0.75) | 4 (2.7-4.3) | 0.79 (0.66-0.94) |
|  | PD-L1: TPS ≥ 1% | > First line, DTX 75 mg/m^2^ Q3W | 343 | 8.5 (7.5-9.8) |  | 4 (3.1-4.2) |  |
|  | PD-L1: TPS ≥ 50% | > First line, PEM 2 mg/kg Q3W | 139 | 14.9 (10.4-NE) | 0.54 (0.38-0.77) | 5.2 (4.1-8.1) | 0.59 (0.44-0.78) |
|  | PD-L1: TPS ≥ 50% | > First line, PEM 10 mg/kg Q3W | 151 | 17.3 (11.8-NE) | 0.5 (0.36-0.7) | 4.1 (3.6-4.3) | 0.59 (0.45-0.78) |
|  | PD-L1: TPS ≥ 50% | > First line, DTX 75 mg/m^2^ Q3W | 152 | 8.2 (6.4-10.7) |  | NR |  |
|  | PD-L1: TPS ≥ 50% | > First line, PEM and DTX, 2 mg/kg Q3W or 10 mg/kg Q3W or 75 mg/m^2^ Q3W | 442 | NR | 0.53 (0.4-0.7) | NR | 0.59 (0.46-0.74) |
|  | PD-L1:  TPS 1%-49% |  | 591 | NR | 0.76 (0.6-0.96) | NR | 1.04 (0.85-1.27) |
|  | PD-L1: TPS ≥ 50% | ≥ 1st line, PEM 2 mg/kg Q3W | NR | 14.9 (10.4-NR) | 0.54 (0.38-0.77) | NR | NR |
|  | PD-L1: TPS ≥ 50% | ≥ 1st line, DTX 75 mg/m^2^ Q3W | NR | 8.2 (6.4-10.7) |  | NR | NR |
|  | PD-L1:  TPS 1%-24% | ≥ 1st line, PEM  2 or 10 mg/kg Q3W | 324 | 9.7 (NR-NR) | NR | 2.6 (NR-NR) | NR |
|  | PD-L1:  TPS 25%-49% |  | 76 | 9.8 (NR-NR) | NR | 2.9 (NR-NR) | NR |
|  | PD-L1:  TPS 50%-74% |  | 106 | 15.8 (NR-NR) | NR | 4.3 (NR-NR) | NR |
|  | PD-L1: TPS ≥ 75% |  | 184 | 16.6 (NR-NR) | NR | 6.2 (NR-NR) | NR |
|  | PD-L1:  TPS 1%-24% | ≥ 1st line, PEM and DTX, 2 mg/kg Q3W or 10 mg/kg Q3W or 75 mg/m^2^ Q3W | 471 | NR | 0.74 (0.56-0.96) | NR | 1.08 (0.86-1.36) |
|  | PD-L1:  TPS 25%-49% |  | 120 | NR | 0.86 (0.51-1.45) | NR | 0.95 (0.6-1.5) |
|  | PD-L1:  TPS 50%-74% |  | 158 | NR | 0.58 (0.36-0.95) | NR | 0.78 (0.52-1.17) |
|  | PD-L1:  TPS ≥ 75% |  | 284 | NR | 0.51 (0.36-0.73) | NR | 0.52 (0.38-0.69) |
|  | PD-L1:  TPS 1%-24% | ≥ First line, DTX 75 mg/m^2^ Q3W | 147 | 8.5 (NR-NR) | NR | 4 (NR-NR) | NR |
|  | PD-L1:  TPS 25%-49% |  | 44 | 9.9 (NR-NR) | NR | 3.8 (NR-NR) | NR |
|  | PD-L1:  TPS 50%-74% |  | 52 | 8.2 (NR-NR) | NR | 4.3 (NR-NR) | NR |
|  | PD-L1: TPS ≥ 75% |  | 100 | 8.2 (NR-NR) | NR | 4 (NR-NR) | NR |
|  | PD-L1: TPS ≥ 50% (archival) |  | 119 | 11.5 (NR-NR) | NA | 3.9 (NR-NR) | NA |
|  | PD-L1: TPS ≥ 50% (new tissue) | ≥ First line, DTX 75 mg/m^2^ Q3W | 171 | NE (NR-NR) | NA | 6.3 (NR-NR) | NA |
|  | PD-L1: TPS ≥ 1% (archival) |  | 300 | 10.5 (NR-NR) | NA | 2.9 (NR-NR) | NA |
|  | PD-L1: TPS ≥ 1% (new tissue) |  | 390 | 12.6 (NR-NR) | NA | 4.1 (NR-NR) | NA |
|  | PD-L1: TPS ≥ 50% (archival) | ≥ First line, PEM and DTX, 2 mg/kg Q3W or 10 mg/kg Q3W or 75 mg/m^2^ Q3W | 184 | NA (NA-NA) | 0.6 (0.4-0.9) | NA | 0.64 (0.45-0.9) |
|  | PD-L1: TPS ≥ 50% (new tissue) | ≥ First line, PEM and DTX, 2 mg/kg Q3W or 10 mg/kg Q3W or 75 mg/m^2^ Q3W | 258 | NA (NA-NA) | 0.44 (0.29-0.66) | NA | 0.54 (0.39-0.75) |
|  | PD-L1: TPS ≥ 1% (archival) |  | 455 | NA (NA-NA) | 0.7 (0.54-0.89) | NA | 0.81 (0.65-1.01) |
|  | PD-L1: TPS ≥ 1% (new tissue) |  | 578 | NA (NA-NA) | 0.64 (0.5-0.83) | NA | 0.86 (0.7-1.07) |
|  | PD-L1: TPS ≥ 50% (archival) | ≥ First line, DTX 75 mg/m^2^ Q3W | 65 | 7.4 (NR-NR) | NA | 4 (NR-NR) | NA |
|  | PD-L1: TPS ≥ 50% (new tissue) |  | 87 | 8.3 (NR-NR) | NA | 4.3 (NR-NR) | NA |
|  | PD-L1: TPS ≥ 1% (archival) |  | 155 | 8.3 (NR-NR) | NA | 3.8 (NR-NR) | NA |
|  | PD-L1: TPS ≥ 1% (new tissue) |  | 188 | 8.6 (NR-NR) | NA | 4.2 (NR-NR) | NA |
| OAK [S-[45-49](#_ENREF_45)] | PD-L1 ≥ 50% | ATEZO | 72 | 20.5 (17.5-NE) | 0.41 (0.27-0.64) | 4.2 (2.9-7) | 0.63 (0.43-0.91) |
|  |  | DTX | 65 | 8.9 (5.6-11.6) |  | 3.3 (2.7-4.2) |  |
|  | PD-L1 ≥ 5% | ATEZO | 129 | 16.3 (13.3-20.1) | 0.67 (0.49-0.90) | 4.1 (2.8-5.3) | 0.76 (0.58-0.99) |
|  |  | DTX | 136 | 10.8 (8.8-12.7) |  | 3.6 (2.8-4.2) |  |
|  | PD-L1 ≥ 1% | ATEZO | 241 | 15.7 (12.6-18.0) | 0.74 (0.58-0.93) | 2.8 (2.6-4.0) | 0.91 (0.74-1.12) |
|  |  | DTX | 222 | 10.3 (8.8-12.0) |  | 4.1 (2.9-4.3) |  |
|  | PD-L1 < 1% | ATEZO | 180 | 12.6 (9.6-15.2) | 0.75 (0.59-0.96) | 2.6 (1.7-2.9) | 1 (0.8-1.25) |
|  |  | DTX | 199 | 8.9 (7.7-11.5) |  | 4 (3.1-4.2) |  |
|  | PD-L1 ≥ 50% (nonsquamous) | ATEZO | 49 | 22.5 (NR) | 0.35 (0.21-0.61) | NR | NR |
|  |  | DTX | 47 | 8.7 (NR) |  | NR | NR |
|  | PD-L1 ≥ 5% (nonsquamous) | ATEZO | 89 | 18.7 (NR) | 0.61 (0.42-0.88) | NR | NR |
|  |  | DTX | 99 | 11.3 (NR) |  | NR | NR |
|  | PD-L1 ≥ 1% (nonsquamous) | ATEZO | 171 | 17.6 (NR) | 0.72 (0.55-0.95) | NR | NR |
|  |  | DTX | 162 | 11.3 (NR) |  | NR | NR |
|  | PD-L1 < 1% (nonsquamous) | ATEZO | 140 | 14 (NR) | 0.75 (0.57-1.0) | NR | NR |
|  |  | DTX | 150 | 11.2 (NR) |  | NR | NR |
|  | PD-L1 ≥ 50% (squamous) | ATEZO | 23 | 17.5 (NR) | 0.57 (0.27-1.2) | NR | NR |
|  |  | DTX | 18 | 11.6 (NR) |  | NR | NR |
|  | PD-L1 ≥ 5% (squamous) | ATEZO | 40 | 10.4 (NR) | 0.76 (0.45-1.29) | NR | NR |
|  |  | DTX | 37 | 9.7 (NR) |  | NR | NR |
|  | PD-L1 ≥ 1% (squamous) | ATEZO | 70 | 9.9 (NR) | 0.71 (0.48-1.06) | NR | NR |
|  |  | DTX | 60 | 8.7 (NR) |  | NR | NR |
|  | PD-L1 < 1% (squamous) | ATEZO | 40 | 7.6 (NR) | 0.82 (0.51-1.32) | NR | NR |
|  |  | DTX | 49 | 7.1 (NR) |  | NR | NR |
|  | PD-L1 ≥ 1% (Japanese) | ATEZO | 11 | 21.3 (15-NE) | 0.81 (0.22-3.05) | 4.2 (2.9-10.2) | 1.18 (0.44-3.16) |
|  |  | DTX | 8 | NE (NE-NE) |  | 5.6 (4.2-8.8) |  |
|  | PD-L1 < 1% (Japanese) | ATEZO | 25 | 20.9 (7.8-NE) | 0.79 (0.36-1.73) | 4 (1.5-4.4) | 1.45 (0.78-2.69) |
|  |  | DTX | 20 | 17 (12-NE) |  | 4.2 (2.9-5.8) |  |
| POPLAR [S-[49-54](#_ENREF_49)] | PD-L1 ≥ 50% | ATEZO | 24 | 15.5 (9.8-NE) | 0.49 (0.22-1.07) | 7.8 (2.7-12.3) | 0.6 (0.31-1.16) |
|  |  | DTX | 23 | 11.1 (6.7-14.4) |  | 3.9 (1.9-5.7) |  |
|  | PD-L1 ≥ 5% | ATEZO | 50 | 15.1 (8.4-NE) | 0.54 (0.33-0.89) | 3.4 (1.4-6.9) | 0.72 (0.47-1.1) |
|  |  | DTX | 55 | 7.4 (6.0-12.5) |  | 2.8 (1.9-3.9) |  |
|  | PD-L1 ≥ 1% | ATEZO | 93 | 15.5 (11-NE) | 0.59 (0.40-0.85) | 2.8 (2.6-5.5) | 0.85 (0.63-1.16) |
|  |  | DTX | 102 | 9.2 (7.3-12.8) |  | 3 (2.8-4.1) |  |
|  | PD-L1 < 1% | ATEZO | 51 | 9.7 (6.7-12) | 1.04 (0.62-1.75) | 1.7 (1.4-4.2) | 1.12 (0.72-1.77) |
|  |  | DTX | 41 | 9.7 (8.6-12) |  | 4.1 (2.7-5.6) |  |
| NCT00730639  Topalian et al. (2012) [S-[55](#_ENREF_55), [56](#_ENREF_56)] | PD-L1 ≥ 5% | NIVO 1.0, 3.0, or 10.0 mg/kg Q2W | 33 | 7.8 (5.6-21.7) | NR | 3.3 (1.8-7.5) | NR |
|  | PD-L1 < 5% |  | 35 | 10.5 (5.2-14.8) | NR | 1.8 (1.7-2.3) | NR |
| NCT01375842 [S-[57](#_ENREF_57), [58](#_ENREF_58)] | PD-L1 < 1% | ATEZO 0.01, 0.03, and 0.1 mg and 0.3, 1, 3, 10, and 20 mg/kg Q3W | 20 | NR | NR | 13 (6-37) | NR |
|  | PD-L1 1 ≤ 5% |  | 13 | NR | NR | 6 (5-43) | NR |
|  | PD-L1 5%-10% |  | 7 | NR | NR | 11 (1-17) | NR |
|  | PD-L1 ≥ 10% |  | 6 | NR | NR | NE (5-NE) | NR |
| Kaderbhai (2017) [S-[62](#_ENREF_62)] | PD-L1 ≥ 1% | NIVO | 33 | NR | 1.4 (0.63-3.09) | NR | 0.97 (0.55-1.69) |
|  | PD-L1 < 1% |  | 33 | NR |  | NR |  |
| Sorensen et al. (2016) [S-[67](#_ENREF_67)] | PD-L1 ≥ 50%, all patients | PEM + platinum-doublet chemotherapy as initial therapy | 51 | 9 (6.4-11.1) | 1.36 (0.9-2.06) | NR | NR |
|  | PD-L1 ≥ 50%, adenocarcinoma |  | NR | 10.9 (6.9-13.3) | 1.31 (0.76-2.27) | NR | NR |
|  | PD-L1 ≥ 50%, squamous-cell carcinoma |  | NR | 7.2 (0.9-10.2) | 3.87 (1.05-14.26) | NR | NR |
|  | PD-L1 1%-49%, all patients |  | 102 | 9.8 (8.2-12.3) | 1.09 (0.76-1.58) | NR | NR |
|  | PD-L1 1%-49%, adenocarcinoma |  | NR | 12.1(8.5-15.0) | 0.84 (0.53-1.34) | NR | NR |
|  | PD-L1 1%-49%, squamous-cell carcinoma |  | NR | 8.8 (6.1-12.2) | 2.36 (0.84-6.63) | NR | NR |
|  | PD-L1 < 1%, all patients |  | 51 | 7.5 (6.4-12.4) | NR | NR | NR |
|  | PD-L1 < 1%, adenocarcinoma |  | NR | 10.7 (7.1-16.4) | NR | NR | NR |
|  | PD-L1 < 1%, squamous-cell carcinoma |  | NR | 19.9 (3.3-NE) | NR | NR | NR |
|  | PD-L1 ≥ 1%, all patients |  | NR | 9.3 (7.8-11.0) | 1.17 (0.83-1.66) | NR | NR |
|  | PD-L1 ≥ 1%, adenocarcinoma |  | NR | 11.1(9.2-13.3) | 0.96 (0.62-1.48) | NR | NR |
|  | PD-L1 ≥ 1%, squamous-cell carcinoma |  | NR | 8.4 (6.6-9.8) | 2.4 (0.87-6.6) | NR | NR |
| Yaghmour (2016) [S-[68](#_ENREF_68)] | PD-1 positive | ≥ First line, NIVO or IPI | 50 overall patients | 7.5 (NR-NR) | 1.53 (0.38-7.58) | NR | NR |
|  | PD-1 negative |  |  | Undefined  (NR-NR) |  | NR | NR |
|  | PD-L1 positive |  |  | 7.5 (NR-NR) | 1.76 (0.5-6.85) | NR | NR |
|  | PD-L1 negative |  |  | Undefined (NR-NR) |  | NR | NR |

ATEZO = atezolizumab; AVE= avelumab; CI = confidence interval; CT = chemotherapy; DTX = docetaxel; DURVA = durvalumab; HR = hazard ratio; IC = immune cell; IFN = interferon; IFN-γ = interferon gamma; IHC = immunohistochemistry; IPI = ipilimumab; mRNA = messenger RNA; NA =not applicable ; NE = not estimable/not reached; NIVO= nivolumab; NR = not reported; OS = overall survival; PD-1 = programmed cell death protein 1; PD-L1 = programmed cell death ligand 1; PEM = pembrolizumab; PFS = progression-free survival; PS = proportion score; Q2W = every 2 weeks; Q3W = every 3 weeks; TC = tumor cell; TPS = tumor proportion score.

Table S3. PD-L1 and TMB: OS and PFS Data in NSCLC

| Trial Acronym | Population | Treatment | No. of Patients | OS | | PFS | |
| --- | --- | --- | --- | --- | --- | --- | --- |
|  |  |  |  | Median (95% CI), Months | HR (95% CI) | Median (95% CI), Months | HR (95% CI) |
| CheckMate 227 [S-[15](#_ENREF_15)] | ≥ 10 mutations per megabase + PD-L1 ≥ 1% | Chemotherapy | 112 | NR | NR | NR | NR (NR-NR) |
|  |  | NIVO + IPI | 101 | NR | NR | 7.1 (5.5-13.5) | 0.75 (0.53-1.07) |
|  |  | NIVO | 102 | NR | NR | 4.2 (2.6-8.3) |  |
|  | ≥ 10 mutations per megabase + PD-L1 < 1% | NIVO + IPI | 38 | NR | NR | NR | 0.48 (0.27-0.85) |
|  |  | Chemotherapy | 48 | NR | NR | NR |  |
|  | ≥ 13 mutations per megabase + PD-L1 ≥ 1% | NIVO | 71 | NR | NR | 4.2 (2.7-8.3) | 0.95 (0.64-1.4) |
|  |  | Chemotherapy | 79 | NR | NR | 5.6 (4.5-7) |  |

CI = confidence interval; HR = hazard ratio; IPI = ipilimumab; NIVO = nivolumab; NR = not reported; NSCLC = non–small-cell lung cancer; OS = overall survival; PD-L1 = programmed cell death ligand 1; PFS = progression-free survival; TMB = tumor mutational burden.

Supplementary Table S4. PD-L1: OS and PFS Data in Melanoma

| Trial Acronym | Population | Treatment | No. of Patients | OS | | PFS | |
| --- | --- | --- | --- | --- | --- | --- | --- |
|  |  |  |  | Median (95% CI), Months | HR (95% CI) | Median (95% CI), Months | HR (95% CI) |
| CheckMate 066 [S-[75](#_ENREF_75)] | PD-L1 ≥ 5% | NIVO 3 mg/kg Q2W | 74 | NE (NE) | NR | NR | NR |
|  | PD-L1 < 5% |  | 128 | NE (NE) | NR | NR | NR |
|  | PD-L1 ≥ 5% | Dacarbazine 100 mg/m^2^ Q3W | 74 | 12.4 (9.2 -NE) | NR | NR | NR |
|  | PD-L1 < 5% |  | 126 | 10.2 (7.6-11.8) | NR | NR | NR |
| CheckMate 066/067/069 [S-[76](#_ENREF_76)] | PD-L1 ≥ 5% | NIVO 1 mg/kg + IPI 3 mg/kg Q3W | 832 (overall patients) | NR | NR | NE | 0.99 (0.66-1.46) |
|  |  | NIVO 3 mg/kg Q2W |  | NR | NR | 22 |  |
|  | PD-L1 < 5% | NIVO 1 mg/kg + IPI 3 mg/kg Q3W |  | NR | NR | 11.1 | 0.7 (0.57-0.87) |
|  |  | NIVO 3 mg/kg Q2W |  | NR | NR | 4.9 |  |
| CheckMate 067 [S-[77-79](#_ENREF_77)] | PD-L1 ≥ 5% | NIVO 3 mg/kg Q2W | 80 | NR | NR | 14 (9.1-NE) | NR |
|  |  | NIVO 1 mg/kg + IPI 3 mg/kg Q3W | 68 | NR | NR | 14 (9.7-NE) | NR |
|  |  | IPI 3 mg/kg Q3W | 75 | NR | NR | 3.9 (2.8-4.2) | NR |
|  | PD-L1 < 5% | NIVO 3 mg/kg Q2W | 208 | NR | NR | 5.3 (2.8-7.1) | NR |
|  |  | NIVO 1 mg/kg + IPI 3 mg/kg Q3W | 210 | NR | NR | 11.2 (8.0-NE) | NR |
|  |  | IPI 3 mg/kg Q3W | 202 | NR | NR | 2.8 (2.8 to NE) | NR |
| KEYNOTE-001 [S-[33-40](#_ENREF_33), [82](#_ENREF_82)] | PD-L1 ≥ 1% | PEM 2 mg/kg Q3W or 10 mg/kg Q2W or Q3W | 344 | 29.9 (24.6-NE) | 0.5 (0.37-0.67) | 5.6 (4.4-8.1) | 0.51 (0.4-0.65) |
|  | PD-L1 < 1% |  | 107 | 12.6 (7-18.5) |  | 2.8 (2.7-2.8) |  |
|  | PD-L1 positive | PEM for measurable disease at baseline | 535 | NR | < 0.001 | NR | NR |
|  | PD-L1 negative |  | 106 | NR |  | NR | NR |
| KEYNOTE-006 [S-[84-86](#_ENREF_84)] | PD-L1 ≥ 1% | PEM 10 mg/kg Q2W | 450 | NR | 0.55 (0.4-0.76) | NR | 0.53 (0.41-0.67) |
|  | PD-L1 < 1% |  | 96 | NR | 0.91 (0.49-1.69) | NR | 0.67 (0.41-1.11) |
|  | PD-L1 ≥ 1% | PEM 10 mg/kg Q3W | 456 | NR | 0.58 (0.42-0.79) | NR | 0.52 (0.4-0.66) |
|  | PD-L1 < 1% |  | 101 | NR | 1.02 (0.56-1.85) | NR | 0.76 (0.47-1.24) |
|  | PD-L1 ≥ 1% | First- or second-line PEM vs. IPI | 671 | NR | 0.56 (0.43-0.74) | NR | 0.52 (0.43-0.64) |
|  | PD-L1 < 1% |  | 150 | NR | 0.94 (0.56-1.6) | NR | 0.83 (0.55-1.26) |
|  | PD-L1 ≥ 1% | First-line PEM vs. IPI | 667 | NR | 0.56 (0.43-0.73) | NR | 0.52 (0.43-0.64) |
|  | PD-L1 < 1% |  | 150 | NR | 0.95 (0.56-1.62) | NR | 0.83 (0.55-1.26) |

CI = confidence interval; IPI= ipilimumab; NE= not estimable/not reached; NIVO = nivolumab; NR= not reported; HR = hazard ratio; OS = overall survival; PEM= pembrolizumab; PD-L1 = programmed cell death ligand 1; PFS = progression-free survival; Q2W = every 2 weeks; Q3W = every 3 weeks.

# References

[S-1] Peters S, Gettinger S, Johnson ML, Janne PA, Garassino MC, Christoph D, et al. Phase II trial of atezolizumab as first-line or subsequent therapy for patients with programmed death-ligand 1-selected advanced non-small-cell lung cancer (BIRCH). J Clin Oncol. 2017 Jun 13;35(24):Jco2016719476. doi: 10.1200/jco.2016.71.9476. <https://www.ncbi.nlm.nih.gov/pubmed/28609226>.

[S-2] Garassino M, Rizvi N, Besse B, Jänne P, Christoph D, Peters S, et al. Atezolizumab as 1l therapy for advanced NSCLC in PD-l1eselected patients: Updated ORR, PFS and OS data from the BIRCH study. J Thorac Oncol. 2017;12(1):S251-S2. <http://www.embase.com/search/results?subaction=viewrecord&from=export&id=L615339132>.

[S-3] Carcereny E, Felip E, Reck M, Patel J, Heist R, Balmanoukian A, et al. Updated efficacy results from the BIRCH study: First-line atezolizumab therapy in PD-L1-selected patients with advanced NSCLC. J Thorac Oncol. 2017 Nov;12(11):S1791-S2. <http://www.embase.com/search/results?subaction=viewrecord&from=export&id=L620147864>.

[S-4] Rizvi NA, Hellmann MD, Brahmer JR, Juergens RA, Borghaei H, Gettinger S, et al. Nivolumab in combination with platinum-based doublet chemotherapy for first-line treatment of advanced non-small-cell lung cancer. J Clin Oncol. 2016 Sep 01;34(25):2969-79. doi: 10.1200/jco.2016.66.9861. <https://www.ncbi.nlm.nih.gov/pubmed/27354481>.

[S-5] Goldman JW, Antonia SJ, Gettinger SN, Borghaei H, Brahmer JR, Ready NE, et al. Nivolumab (N) plus ipilimumab (I) as first-line (1L) treatment for advanced (adv) NSCLC: 2-yr OS and long-term outcomes from CheckMate 012. J Clin Oncol. 2017 May 20;35(15). doi: 10.1200/JCO.2017.35.15_suppl.9093. <http://www.embase.com/search/results?subaction=viewrecord&from=export&id=L617434994>.

[S-6] Brahmer J, Reckamp KL, Baas P, Crino L, Eberhardt WE, Poddubskaya E, et al. Nivolumab versus docetaxel in advanced squamous-cell non-small-cell lung cancer. N Engl J Med. 2015 Jul 09;373(2):123-35. doi: 10.1056/NEJMoa1504627. <https://www.ncbi.nlm.nih.gov/pubmed/26028407>.

[S-7] Eberhardt WEE, Borghaei H, Brahmer JR, Horn L, Ready N, Steins M, et al. CheckMate 017 and 057 studies of nivolumab vs docetaxel in patients with advanced NSCLC: 2-year-update and exploratory cytokine profile analyses. Oncol Res Treat. 2016 Oct;39:33. doi: 10.1159/000449050. <http://www.embase.com/search/results?subaction=viewrecord&from=export&id=L613153451http://dx.doi.org/10.1159/000449050>.

[S-8] Font EF, Gettinger S, Burgio MA, Antonia S, Holgado E, Spigel D, et al. Three-year follow-up from CheckMate 017/057: Nivolumab versus docetaxel in patients with previously treated advanced non-small cell lung cancer (NSCLC). Ann Oncol. 2017 Sep;28(Supplement 5):462. doi: 10.1093/annonc/mdx380.004.

[S-9] Carbone DP, Reck M, Paz-Ares L, Creelan B, Horn L, Steins M, et al. First-line nivolumab in stage IV or recurrent non-small-cell lung cancer. N Engl J Med. 2017 Jun 22;376(25):2415-26. doi: 10.1056/NEJMoa1613493. <http://www.embase.com/search/results?subaction=viewrecord&from=export&id=L616878978http://dx.doi.org/10.1056/NEJMoa1613493>.

[S-10] Socinski M, Creelan B, Horn L, Reck M, Paz-Ares L, Steins M, et al. PR CheckMate 026: a phase 3 trial of nivolumab vs investigator's choice (IC) of platinum-based doublet chemotherapy (PT-DC) as first-line therapy for stage iv/recurrent programmed death ligand 1 (PD-L1)-positive NSCLC. Ann Oncol. 2016;27(suppl_6). doi: 10.1093/annonc/mdw435.39. <http://www.embase.com/search/results?subaction=viewrecord&from=export&id=L613912423http://dx.doi.org/10.1093/annonc/mdw435.39>.

[S-11] Borghaei H, Paz-Ares L, Horn L, Spigel DR, Steins M, Ready NE, et al. Nivolumab versus docetaxel in advanced nonsquamous non-small-cell lung cancer. N Engl J Med. 2015 Oct 22;373(17):1627-39. doi: 10.1056/NEJMoa1507643. <https://www.ncbi.nlm.nih.gov/pubmed/26412456>.

[S-12] Peters S, Cappuzzo F, Horn L, Paz-Ares L, Borghaei H, Barlesi F, et al. Analysis of early survival in patients with advanced non-squamous NSCLC treated with nivolumab vs docetaxel in CheckMate 057. J Thorac Oncol. 2017 Jan;12(1):S253. <http://www.embase.com/search/results?subaction=viewrecord&from=export&id=L615339232>.

[S-13] Paz-Ares L, Horn L, Borghaei H, Spigel DR, Steins M, Ready N, et al. Phase III, randomized trial (CheckMate 057) of nivolumab (NIVO) versus docetaxel (DOC) in advanced non-squamous cell (non-SQ) non-small cell lung cancer (NSCLC). J Clin Oncol. 2015;33(15_suppl):LBA109-LBA. doi: 10.1200/jco.2015.33.15_suppl.lba109. <http://ascopubs.org/doi/abs/10.1200/jco.2015.33.15_suppl.lba109>.

[S-14] Rizvi NA, Mazieres J, Planchard D, Stinchcombe TE, Dy GK, Antonia SJ, et al. Activity and safety of nivolumab, an anti-PD-1 immune checkpoint inhibitor, for patients with advanced, refractory squamous non-small-cell lung cancer (CheckMate 063): a phase 2, single-arm trial. Lancet Oncol. 2015 Mar;16(3):257-65. doi: 10.1016/s1470-2045(15)70054-9. <https://www.ncbi.nlm.nih.gov/pubmed/25704439>.

[S-15] Hellmann MD, Ciuleanu TE, Pluzanski A, Lee JS, Otterson GA, Audigier-Valette C, et al. Nivolumab plus ipilimumab in lung cancer with a high tumor mutational burden. N Engl J Med. 2018 Apr 16;378(22):2093-104. doi: 10.1056/NEJMoa1801946. <https://www.ncbi.nlm.nih.gov/pubmed/29658845>.

[S-16] Higgs BW, Robbins PB, Blake-Haskins JA, Zhu W, Morehouse C, Brohawn PZ, et al. High tumoral IFNγ mRNA, PD-L1 protein, and combined IFNγ mRNA/PD-L1 protein expression associates with response to durvalumab (anti-PD-L1) monotherapy in NSCLC patients. Eur J Cancer. 2015;51:S717. <http://www.embase.com/search/results?subaction=viewrecord&from=export&id=L72068631>.

[S-17] Streicher K, Morehouse C, Sebastian Y, Kuziora M, Higgs BW, Ranade K. Gene expression analysis of tumor biopsies from a trial of durvalumab to identify subsets of NSCLC with shared immune pathways. J Clin Oncol. 2017 May 20;35(15). doi: 10.1200/JCO.2017.35.15_suppl.3041. <http://www.embase.com/search/results?subaction=viewrecord&from=export&id=L617434960>.

[S-18] Balmanoukian AS, Antonia SJ, Hwu WJ, Hamid O, Gutierrez M, Jamal R, et al. Updated safety and clinical activity of durvalumab monotherapy in previously treated patients with stage IIIB/IV NSCLC. Journal of Clinical Oncology. 2017 May 20;35(15). doi: 10.1200/JCO.2017.35.15_suppl.9085. <http://www.embase.com/search/results?subaction=viewrecord&from=export&id=L617435659>.

[S-19] Higgs BW, Morehouse C, Streicher K, Rebelatto MC, Steele K, Jin X, et al. Relationship of baseline tumoral IFN mRNA and PD-L1 protein expression to overall survival in durvalumab-treated NSCLC patients. J Clin Oncol. 2016 May 20;34(15). doi: 10.1200/JCO.2016.34.15_suppl.3036. <http://www.embase.com/search/results?subaction=viewrecord&from=export&id=L611751931>.

[S-20] Antonia S, Rizvi N, Brahmer J, Ou SH, Khleif SN, Hwu WJ, et al. Safety and clinical activity of durvalumab (MEDI4736), an anti-programmed cell death ligand-1 (PD-L1) antibody, in patients with nonsmall cell lung cancer (NSCLC). Cancer Immunol Res. 2016;4(1). doi: 10.1158/2326-6074.cricimteatiaacr15-a047. <http://www.embase.com/search/results?subaction=viewrecord&from=export&id=L613321787http://dx.doi.org/10.1158/2326-6074.CRICIMTEATIAACR15-A047>.

[S-21] Antonia SJ, Brahmer JR, Khleif S, Balmanoukian AS, Ou SHI, Gutierrez M, et al. Phase 1/2 study of the safety and clinical activity of durvalumab in patients with non-small cell lung cancer (NSCLC). Annals of Oncology. 2016 Oct 1;27. doi: 10.1093/annonc/mdw383.16. <http://www.embase.com/search/results?subaction=viewrecord&from=export&id=L613912187http://dx.doi.org/10.1093/annonc/mdw383.16>.

[S-22] Rizvi NA, Brahmer JR, Ou S-HI, Segal NH, Khleif S, Hwu W-J, et al. Safety and clinical activity of MEDI4736, an anti-programmed cell death-ligand 1 (PD-L1) antibody, in patients with non-small cell lung cancer (NSCLC). J Clin Oncol. 2015 May 20;33(15_suppl):8032-. doi: 10.1200/jco.2015.33.15_suppl.8032. <http://ascopubs.org/doi/abs/10.1200/jco.2015.33.15_suppl.8032>.

[S-23] Gulley JL, Rajan A, Spigel DR, Iannotti N, Chandler J, Wong DJL, et al. Avelumab for patients with previously treated metastatic or recurrent non-small-cell lung cancer (JAVELIN Solid Tumor): dose-expansion cohort of a multicentre, open-label, phase 1b trial. Lancet Oncol. 2017 May;18(5):599-610. doi: 10.1016/s1470-2045(17)30240-1. <https://www.ncbi.nlm.nih.gov/pubmed/28373005>.

[S-24] Gulley JL, Spigel DR, Kelly K, Aisner J, Chand VK, Koenig A, et al. Exposure-response and PD-L1 expression analysis of second-line avelumab in patients with advanced NSCLC: Data from the JAVELIN Solid Tumor trial. J Clin Oncol. 2017 May 20;35(15). doi: 10.1200/JCO.2017.35.15_suppl.9086. <http://www.embase.com/search/results?subaction=viewrecord&from=export&id=L617435399>.

[S-25] Apolo AB, Ellerton JA, Infante JR, Agrawal M, Gordon MS, Aljumaily R, et al. Updated efficacy and safety of avelumab in metastatic urothelial carcinoma (mUC): Pooled analysis from 2 cohorts of the phase 1b Javelin solid tumor study. J Clin Oncol. 2017 May 20;35(15). doi: 10.1200/JCO.2017.35.15_suppl.4528. <http://www.embase.com/search/results?subaction=viewrecord&from=export&id=L617435213>.

[S-26] Verschraegen CF, Chen F, Spigel DR, Iannotti N, McClay EF, Redfern CH, et al. Avelumab (MSB0010718C; anti-PD-L1) as a first-line treatment for patients with advanced NSCLC from the JAVELIN Solid Tumor phase 1b trial: Safety, clinical activity, and PD-L1 expression. Journal of Clinical Oncology. 2016;34. <http://www.embase.com/search/results?subaction=viewrecord&from=export&id=L611752784>.

[S-27] Apolo AB, Infante JR, Hamid O, Patel MR, Wang D, Kelly K, et al. Avelumab (MSB0010718C; anti-PD-L1) in patients with metastatic urothelial carcinoma from the JAVELIN solid tumor phase 1b trial: Analysis of safety, clinical activity, and PD-L1 expression. Journal of Clinical Oncology. 2016;34. <http://www.embase.com/search/results?subaction=viewrecord&from=export&id=L611752797>.

[S-28] Hassan R, Thomas A, Patel MR, Nemunaitis JJ, Bennouna J, Powderly JD, et al. Avelumab (MSB0010718C; anti-PD-LI) in patients with advanced unresectable mesothelioma from the JAVELIN solid tumor phase Ib trial: Safety, clinical activity, and PD-L1 expression. J Clin Oncol. 2016;34. <http://www.embase.com/search/results?subaction=viewrecord&from=export&id=L611752983>.

[S-29] Disis ML, Patel MR, Pant S, Hamilton EP, Lockhart AC, Kelly K, et al. Avelumab (MSB0010718C; anti-PD-L1) in patients with recurrent/refractory ovarian cancer from the JAVELIN Solid Tumor phase Ib trial: Safety and clinical activity. J Clin Oncol. 2016;34. <http://www.embase.com/search/results?subaction=viewrecord&from=export&id=L611753805>.

[S-30] Gulley JL, Spigel D, Kelly K, Chandler JC, Rajan A, Hassan R, et al. Avelumab (MSB0010718C), an anti-PD-L1 antibody, in advanced NSCLC patients: A phase 1b, open-label expansion trial in patients progressing after platinum-based chemotherapy. J Clin Oncol. 2015 May 20;33(15_suppl):8034-. doi: 10.1200/jco.2015.33.15_suppl.8034. <http://ascopubs.org/doi/abs/10.1200/jco.2015.33.15_suppl.8034>.

[S-31] Apolo AB, Ellerton J, Infente J, Agarwal M, Gordon MS, Aljumaily R, et al. Avelumab treatment of metastatic urothelial carcinoma (mUC) in the phase 1b JAVELIN solid Tumor study: updated analysis with ≥6 months of follow-up in all patients. Ann Oncol. 2017;28(Supplement 5):300.

[S-32] Le Tourneau C, Zarwan C, Hoimes C, Wong DJ, Bauer S, Wermke M, et al. Avelumab in patients with metastatic adrenocortical carcinoma (mACC): Results from the JAVELIN solid tumor trial. Ann Oncol. 2017;28(Supplement 5):324.

[S-33] Garon EB, Rizvi NA, Hui R, Leighl N, Balmanoukian AS, Eder JP, et al. Pembrolizumab for the treatment of non-small-cell lung cancer. N Engl J Med. 2015 May 21;372(21):2018-28. doi: 10.1056/NEJMoa1501824. <https://www.ncbi.nlm.nih.gov/pubmed/25891174>.

[S-34] Daud AI, Wolchok JD, Robert C, Hwu WJ, Weber JS, Ribas A, et al. Programmed death-ligand 1 expression and response to the anti-programmed death 1 antibody pembrolizumab in melanoma. J Clin Oncol. 2016 Dec;34(34):4102-9. doi: 10.1200/jco.2016.67.2477. <https://www.ncbi.nlm.nih.gov/pubmed/27863197>.

[S-35] Hui R, Garon EB, Goldman JW, Leighl NB, Hellmann MD, Patnaik A, et al. Pembrolizumab as first-line therapy for patients with PD-L1-positive advanced non-small cell lung cancer: a phase 1 trial. Ann Oncol. 2017 Apr 01;28(4):874-81. doi: 10.1093/annonc/mdx008. <https://www.ncbi.nlm.nih.gov/pubmed/28168303>.

[S-36] Ribas A, Hamid O, Daud A, Hodi FS, Wolchok JD, Kefford R, et al. Association of Pembrolizumab With Tumor Response and Survival Among Patients With Advanced Melanoma. JAMA. 2016 Apr 19;315(15):1600-9. doi: 10.1001/jama.2016.4059. <https://www.ncbi.nlm.nih.gov/pubmed/27092830>.

[S-37] Leighl NB, Hellmann MD, Hui R, Costa EC, Felip E, Ahn MJ, et al. KEYNOTE-001: 3-year overall survival for patients with advanced NSCLC treated with pembrolizumab. J Clin Oncol. 2017;35(15). <http://www.embase.com/search/results?subaction=viewrecord&from=export&id=L617388701>.

[S-38] Dong ZY, Zhong WZ, Zhang XC, Su J, Xie Z, Liu SY, et al. Potential predictive value of TP53 and KRAS mutation status for response to PD-1 blockade immunotherapy in lung adenocarcinoma. Clin Cancer Res. 2017 Jun 15;23(12):3012-24. doi: 10.1158/1078-0432.ccr-16-2554. <http://www.embase.com/search/results?subaction=viewrecord&from=export&id=L616791879http://dx.doi.org/10.1158/1078-0432.CCR-16-2554>.

[S-39] Hui R, Gandhi L, Costa EC, Felip E, Ahn MJ, Eder JP, et al. Long-term OS for patients with advanced NSCLC enrolled in the KEYNOTE-001 study of pembrolizumab (pembro). J Clin Oncol. 2016 May 20;34(15). doi: 10.1200/JCO.2016.34.15_suppl.9026. <http://www.embase.com/search/results?subaction=viewrecord&from=export&id=L611752290>.

[S-40] Rizvi NA, Garon EB, Leighl N, Hellmann MD, Patnaik A, Gandhi L, et al. Optimizing PDL1 as a biomarker of response with pembrolizumab (pembro; MK-3475) as first-line therapy for PDL1-positive metastatic non-small cell lung cancer (NSCLC): Updated data from KEYNOTE-001. J Clin Oncol. 2015;33(15). <http://www.embase.com/search/results?subaction=viewrecord&from=export&id=L72014685>.

[S-41] Herbst RS, Baas P, Kim DW, Felip E, Perez-Gracia JL, Han JY, et al. Pembrolizumab versus docetaxel for previously treated, PD-L1-positive, advanced non-small-cell lung cancer (KEYNOTE-010): a randomised controlled trial. Lancet. 2016 Apr 09;387(10027):1540-50. doi: 10.1016/s0140-6736(15)01281-7. <https://www.ncbi.nlm.nih.gov/pubmed/26712084>.

[S-42] Huang M, Pellissier J, Burke T, Xu R. Implications of implementation of a PDL1 biomarker-based strategy for treatment of advanced NSCLC. J Thorac Oncol. 2017;12(1):S424. <http://www.embase.com/search/results?subaction=viewrecord&from=export&id=L615338903>.

[S-43] Baas P, Garon EB, Herbst RS, Felip E, Perez-Gracia JL, Han JY, et al. Relationship between level of PD-L1 expression and outcomes in the KEYNOTE-010 study of pembrolizumab vs docetaxel for previously treated, PD-Ll-Positive NSCLC. J Clin Oncol. 2016;34. <http://www.embase.com/search/results?subaction=viewrecord&from=export&id=L611751814>.

[S-44] Herbst RS, Baas P, Perez-Gracia JL, Felip E, Kim DW, Han JY, et al. Archival vs new tumor samples for assessing PD-L1 expression in the KEYNOTE-010 study of pembrolizumab (pembro) vs docetaxel (doce) for previously treated advanced NSCLC. J Clin Oncol. 2016 May 20;34(15). doi: 10.1200/JCO.2016.34.15_suppl.3030. <http://www.embase.com/search/results?subaction=viewrecord&from=export&id=L611754192>.

[S-45] Rittmeyer A, Barlesi F, Waterkamp D, Park K, Ciardiello F, von Pawel J, et al. Atezolizumab versus docetaxel in patients with previously treated non-small-cell lung cancer (OAK): a phase 3, open-label, multicentre randomised controlled trial. Lancet. 2017 Jan 21;389(10066):255-65. doi: 10.1016/s0140-6736(16)32517-x. <http://www.embase.com/search/results?subaction=viewrecord&from=export&id=L613977540http://dx.doi.org/10.1016/S0140-6736(16)32517-X>.

[S-46] Gadgeel S, Ciardiello F, Rittmeyer A, Barlesi F, Cortinovis D, Barrios C, et al. OAK, a randomized ph III study of atezolizumab vs docetaxel in patients with advanced NSCLC: results from subgroup analyses. J Thorac Oncol. 2017;12(1):S9-S10. <http://www.embase.com/search/results?subaction=viewrecord&from=export&id=L615338391>.

[S-47] Barlesi F, Park K, Ciardiello F, Von Pawel J, Gadgeel S, Hida T, et al. PR primary analysis from OAK, a randomized phase III study comparing atezolizumab with docetaxel in 2L/3L NSCLC. Ann Oncol. 2016;27(suppl_6). doi: 10.1093/annonc/mdw435.43. <http://www.embase.com/search/results?subaction=viewrecord&from=export&id=L613912347http://dx.doi.org/10.1093/annonc/mdw435.43>.

[S-48] Hida T, Kaji R, Satouchi M, Ikeda N, Horiike A, Nokihara H, et al. Atezolizumab in Japanese patients with previously treated advanced non-small-cell lung cancer: a subgroup analysis of the phase 3 OAK study. Clin Lung Cancer. 2018 Jul;19(4):e405-e15. doi: 10.1016/j.cllc.2018.01.004. <http://www.embase.com/search/results?subaction=viewrecord&from=export&id=L621079981> <http://dx.doi.org/10.1016/j.cllc.2018.01.004>

<https://www.sciencedirect.com/science/article/pii/S1525730418300160?via%3Dihub>.

[S-49] Gandara DR, Kowanetz M, Mok TSK, Rittmeyer A, Fehrenbacher L, Fabrizio D, et al. Blood-based biomarkers for cancer immunotherapy: tumor mutational burden in blood (bTMB) is associated with improved atezolizumab (atezo) efficacy in 2L1 NSCLC (POPLAR and OAK). Ann Oncol. 2017;28:v460. <http://www.embase.com/search/results?subaction=viewrecord&from=export&id=L619622662>.

[S-50] Fehrenbacher L, Spira A, Ballinger M, Kowanetz M, Vansteenkiste J, Mazieres J, et al. Atezolizumab versus docetaxel for patients with previously treated non-small-cell lung cancer (POPLAR): a multicentre, open-label, phase 2 randomised controlled trial. Lancet. 2016 Apr 30;387(10030):1837-46. doi: 10.1016/s0140-6736(16)00587-0. <https://www.ncbi.nlm.nih.gov/pubmed/26970723>.

[S-51] Smith DA, Vansteenkiste JF, Fehrenbacher L, Park K, Mazieres J, Rittmeyer A, et al. Updated survival and biomarker analyses of a randomized phase II study of atezolizumab vs docetaxel in 2L/3L NSCLC (POPLAR). J Clin Oncol. 2016 May 20;34(15). doi: 10.1200/JCO.2016.34.15_suppl.9028. <http://www.embase.com/search/results?subaction=viewrecord&from=export&id=L611752383>.

[S-52] Mazieres J, Fehrenbacher L, Rittmeyer A, Spira AI, Park K, Smith DA, et al. Non-classical response measured by immune-modified RECIST and post-progression treatment effects of atezolizumab in 2L/3L NSCLC: results from the randomized phase II study POPLAR. J Clin Oncol. 2016 May 20;34(15). doi: 10.1200/JCO.2016.34.15_suppl.9032. <http://www.embase.com/search/results?subaction=viewrecord&from=export&id=L611752613>.

[S-53] Vansteenkiste J, Fehrenbacher L, Spira AI, Mazieres J, Park K, Smith D, et al. Atezolizumab monotherapy vs docetaxel in 2L/3L non-small cell lung cancer: primary analyses for efficacy, safety and predictive biomarkers from a randomized phase II study (POPLAR). Eur J Cancer. 2015 Sep;51:S716-S7. doi: Doi 10.1016/S0959-8049(15)30072-1. <http://www.embase.com/search/results?subaction=viewrecord&from=export&id=L72068630>.

[S-54] Spira AI, Park K, Mazières J, Vansteenkiste JF, Rittmeyer A, Ballinger M, et al. Efficacy, safety and predictive biomarker results from a randomized phase II study comparing MPDL3280A vs docetaxel in 2L/3L NSCLC (POPLAR). J Clin Oncol. 2015 May 20;33(15). <http://www.embase.com/search/results?subaction=viewrecord&from=export&id=L72014669>.

[S-55] Topalian SL, Hodi FS, Brahmer JR, Gettinger SN, Smith DC, McDermott DF, et al. Safety, activity, and immune correlates of anti-PD-1 antibody in cancer. N Engl J Med. 2012 Jun 28;366(26):2443-54. doi: 10.1056/NEJMoa1200690. <https://www.ncbi.nlm.nih.gov/pubmed/22658127>.

[S-56] Gettinger SN, Horn L, Gandhi L, Spigel DR, Antonia SJ, Rizvi NA, et al. Overall Survival and Long-Term Safety of Nivolumab (Anti-Programmed Death 1 Antibody, BMS-936558, ONO-4538) in Patients With Previously Treated Advanced Non-Small-Cell Lung Cancer. J Clin Oncol. 2015 Jun 20;33(18):2004-12. doi: 10.1200/jco.2014.58.3708. <https://www.ncbi.nlm.nih.gov/pubmed/25897158>.

[S-57] Herbst RS, Soria JC, Kowanetz M, Fine GD, Hamid O, Gordon MS, et al. Predictive correlates of response to the anti-PD-L1 antibody MPDL3280A in cancer patients. Nature. 2014 Nov 27;515(7528):563-7. doi: 10.1038/nature14011. <https://www.ncbi.nlm.nih.gov/pubmed/25428504>.

[S-58] Horn L, Spigel DR, Gettinger SN, Antonia SJ, Gordon MS, Herbst RS, et al. Clinical activity, safety and predictive biomarkers of the engineered antibody MPDL3280A (anti-PDL1) in non-small cell lung cancer (NSCLC): Update from a phase Ia study. J Clin Oncol. 2015 May 20;33(15). <http://www.embase.com/search/results?subaction=viewrecord&from=export&id=L72014688>.

[S-59] Bagley SJ, Kothari S, Aggarwal C, Bauml JM, Alley EW, Evans TL, et al. Pretreatment neutrophil-to-lymphocyte ratio as a marker of outcomes in nivolumab-treated patients with advanced non-small-cell lung cancer. Lung Cancer. 2017 Apr;106:1-7. doi: 10.1016/j.lungcan.2017.01.013. <https://www.ncbi.nlm.nih.gov/pubmed/28285682>.

[S-60] De Castro AM, Navarro A, Perez SC, Martinez A, Pardo N, Hernando A, et al. Lactate dehydrogenase (LDH) as a surrogate biomarker to checkpoint-inhibitors for patient with advanced nonesmall-cell lung cancer (NSCLC). J Thorac Oncol. 2017;12(1):S1313-S4. <http://www.embase.com/search/results?subaction=viewrecord&from=export&id=L615339166>.

[S-61] Gettinger SN, Hellmann MD, Shepherd FA, Antonia SJ, Brahmer J, Chow LQ, et al. First-line monotherapy with nivolumab (NIVO) in advanced non-small cell lung cancer (NSCLC): Safety, efficacy, and biomarker analyses. Eur J Cancer. 2015 Sep;51:S632. doi: Doi 10.1016/S0959-8049(16)31737-3. <http://www.embase.com/search/results?subaction=viewrecord&from=export&id=L72068419>.

[S-62] Kaderbhai CG, Richard C, Fumet JD, Aarnink A, Ortiz-Cuaran S, Pérol M, et al. Response to first line chemotherapy regimen is associated with efficacy of nivolumab in non-small-cell lung cancer. OncoImmunology. 2017;6(9):e1339856. doi: 10.1080/2162402x.2017.1339856. <http://www.embase.com/search/results?subaction=viewrecord&from=export&id=L617375514http://dx.doi.org/10.1080/2162402X.2017.1339856>.

[S-63] Nomizo T, Ozasa H, Tsuji T, Funazo T, Yasuda Y, Yoshida H, et al. Clinical Impact of Single Nucleotide Polymorphism in PD-L1 on Response to Nivolumab for Advanced Non-Small-Cell Lung Cancer Patients. Sci Rep. 2017 Mar 23;7:45124. doi: 10.1038/srep45124. <https://www.ncbi.nlm.nih.gov/pubmed/28332580>.

[S-64] Pabla S, Dy G, Nesline M, Gandhi S, Pandey M, Ernstoff MC, et al. The inflamed phenotype in PD-L1 negative non-small cell lung cancer (NSCLC) and response to checkpoint inhibitors. J Immunol. 2017;198(1). <http://www.embase.com/search/results?subaction=viewrecord&from=export&id=L617354833>.

[S-65] Roach C, Zhang N, Corigliano E, Jansson M, Toland G, Ponto G, et al. Development of a Companion Diagnostic PD-L1 Immunohistochemistry Assay for Pembrolizumab Therapy in Non-Small-cell Lung Cancer. Appl Immunohistochem Mol Morphol. 2016 Jul;24(6):392-7. doi: 10.1097/pai.0000000000000408. <https://www.ncbi.nlm.nih.gov/pubmed/27333219>.

[S-66] Sabari JK, Montecalvo J, Chen R, Dienstag JA, Mrad C, Bergagnini I, et al. PD-L1 expression and response to immunotherapy in patients with MET exon 14-altered non-small cell lung cancers (NSCLC). J Clin Oncol. 2017 May 20;35(15). doi: 10.1200/JCO.2017.35.15_suppl.8512. <http://www.embase.com/search/results?subaction=viewrecord&from=export&id=L617388548>.

[S-67] Sorensen SF, Zhou W, Dolled-Filhart M, Georgsen JB, Wang Z, Emancipator K, et al. PD-L1 expression and survival among patients with advanced non–small cell lung cancer treated with chemotherapy. Transl Oncol. 2016 Feb;9(1):64-9. doi: 10.1016/j.tranon.2016.01.003. <http://www.embase.com/search/results?subaction=viewrecord&from=export&id=L610842013http://dx.doi.org/10.1016/j.tranon.2016.01.003>.

[S-68] Yaghmour G, Pandey M, Ireland C, Patel K, Nunnery S, Powell D, et al. Role of genomic instability in immunotherapy with checkpoint inhibitors. Anticancer Res. 2016 Aug;36(8):4033-8. <http://www.embase.com/search/results?subaction=viewrecord&from=export&id=L612866017>.

[S-69] Velcheti V. Prospective clinical evaluation of blood-based tumor mutational burden (bTMB) as a predictive biomarker for atezolizumab (atezo) in 1L non-small cell lung cancer (NSCLC): Interim B-F1RST results. The American Society of Clinical Oncology. Chicago, Illinois; 2018.

[S-70] Antonia SJ, Lopez-Martin JA, Bendell J, Ott PA, Taylor M, Eder JP, et al. Nivolumab alone and nivolumab plus ipilimumab in recurrent small-cell lung cancer (CheckMate 032): a multicentre, open-label, phase 1/2 trial. Lancet Oncol. 2016 Jul;17(7):883-95. doi: 10.1016/s1470-2045(16)30098-5. <https://www.ncbi.nlm.nih.gov/pubmed/27269741>.

[S-71] Hellmann M, Antonia S, Ponce S, Ott P, Calvo E, Taylor M, et al. Nivolumab alone or with ipilimumab in recurrent small cell lung cancer (SCLC): 2-year survival and updated analyses from the checkmate 032 trial. J Thorac Oncol. 2017;12(1):S393-S4. <http://www.embase.com/search/results?subaction=viewrecord&from=export&id=L615339448>.

[S-72] Ji RR, Chasalow SD, Wang L, Hamid O, Schmidt H, Cogswell J, et al. An immune-active tumor microenvironment favors clinical response to ipilimumab. Cancer Immunol Immunother. 2012 Jul;61(7):1019-31. doi: 10.1007/s00262-011-1172-6. <https://www.ncbi.nlm.nih.gov/pubmed/22146893>.

[S-73] Wolchok JD, Kluger H, Callahan MK, Postow MA, Rizvi NA, Lesokhin AM, et al. Nivolumab plus ipilimumab in advanced melanoma. N Engl J Med. 2013 Jul 11;369(2):122-33. doi: 10.1056/NEJMoa1302369. <https://www.ncbi.nlm.nih.gov/pubmed/23724867>.

[S-74] Weber JS, D'Angelo SP, Minor D, Hodi FS, Gutzmer R, Neyns B, et al. Nivolumab versus chemotherapy in patients with advanced melanoma who progressed after anti-CTLA-4 treatment (CheckMate 037): a randomised, controlled, open-label, phase 3 trial. Lancet Oncol. 2015 Apr;16(4):375-84. doi: 10.1016/s1470-2045(15)70076-8. <https://www.ncbi.nlm.nih.gov/pubmed/25795410>.

[S-75] Robert C, Long GV, Brady B, Dutriaux C, Maio M, Mortier L, et al. Nivolumab in previously untreated melanoma without BRAF mutation. N Engl J Med. 2015 Jan 22;372(4):320-30. doi: 10.1056/NEJMoa1412082. <https://www.ncbi.nlm.nih.gov/pubmed/25399552>.

[S-76] Long G, Larkin J, Ascierto P, Hodi S, Rutkowski P, Chiarion-Selini V, et al. PD-L1 expression as a biomarker for nivolumab (NIVO) plus ipilimumab (IPI) and nivo alone in advanced melanoma (MEL): a pooled analysis. Asia Pac J Clin Oncol. 2016;12:126. <http://www.embase.com/search/results?subaction=viewrecord&from=export&id=L613440422>.

[S-77] Larkin J, Chiarion-Sileni V, Gonzalez R, Grob JJ, Cowey CL, Lao CD, et al. Combined nivolumab and ipilimumab or monotherapy in untreated melanoma. N Engl J Med. 2015 Jul 2;373(1):23-34. doi: 10.1056/NEJMoa1504030. <https://www.nejm.org/doi/full/10.1056/NEJMoa1504030>.

[S-78] Wolchok JD, Chiarion-Sileni V, Gonzalez R, Rutkowski P, Grob JJ, Cowey CL, et al. Updated results from a phase III trial of nivolumab (NIVO) combined with ipilimumab (IPI) in treatment-naive patients (pts) with advanced melanoma (MEL) (CheckMate 067). J Clin Oncol. 2016 May 20;34(15). doi: 10.1200/JCO.2016.34.15_supll.9505. <http://www.embase.com/search/results?subaction=viewrecord&from=export&id=L611753541>.

[S-79] Wolchok JD, Chiarion-Sileni V, Gonzalez R, Rutkowski P, Grob JJ, Cowey CL, et al. Updated results from a phase 3 trial of nivolumab (NIVO) combined with ipilimumab (IPI) in treatment-naive patients with advanced melanoma (MEL) (checkmate 067). Asia Pac J Clin Oncol. 2016 Nov;12:127-8. <http://www.embase.com/search/results?subaction=viewrecord&from=export&id=L613440454>.

[S-80] Postow MA, Chesney J, Pavlick AC, Robert C, Grossmann K, McDermott D, et al. Nivolumab and Ipilimumab versus Ipilimumab in Untreated Melanoma. N Engl J Med. 2015 May 21;372(21):2006-17. doi: 10.1056/NEJMoa1414428. <https://www.nejm.org/doi/full/10.1056/NEJMoa1414428>.

[S-81] Homicsko K, Cuendet MA, Mlynska A, Moura B, Horak C, Hanahan D, et al. Exploratory analysis of multiprotein serum predictors at baseline of progression-free survival of ipilimumab or ipilimumab and nivolumab in the CheckMate-069 study. J Clin Oncol. 2017 May 20;35(15). doi: 10.1200/JCO.2017.35.15_suppl.9571. <http://www.embase.com/search/results?subaction=viewrecord&from=export&id=L617435738>.

[S-82] Joseph RW, Elassaiss-Schaap J, Kefford RF, Hwu WJ, Wolchok JD, Joshua AM, et al. Baseline Tumor Size Is an Independent Prognostic Factor for Overall Survival in Patients With Melanoma Treated With Pembrolizumab. Clin Cancer Res. 2018 Apr 23;24(20):4960-7. doi: 10.1158/1078-0432.ccr-17-2386. <http://clincancerres.aacrjournals.org/content/clincanres/early/2018/04/21/1078-0432.CCR-17-2386.full.pdf>.

[S-83] Ribas A, Puzanov I, Dummer R, Schadendorf D, Hamid O, Robert C, et al. Pembrolizumab versus investigator-choice chemotherapy for ipilimumab-refractory melanoma (KEYNOTE-002): a randomised, controlled, phase 2 trial. Lancet Oncol. 2015 Aug;16(8):908-18. doi: 10.1016/s1470-2045(15)00083-2. <https://www.ncbi.nlm.nih.gov/pubmed/26115796>.

[S-84] Robert C, Schachter J, Long GV, Arance A, Grob JJ, Mortier L, et al. Pembrolizumab versus ipilimumab in advanced melanoma. N Engl J Med. 2015 Jun 25;372(26):2521-32. doi: 10.1056/NEJMoa1503093. <https://www.ncbi.nlm.nih.gov/pubmed/25891173>.

[S-85] Carlino M, Emancipator K, Ibrahim N, Zhou H, Schadendorf D, Ribas A, et al. PD-l1 expression and efficacy in patients treated with pembrolizumab vs ipilimumab for advanced melanoma In KEYNOTE-006. Asia Pac J Clin Oncol. 2016;12:44. <http://www.embase.com/search/results?subaction=viewrecord&from=export&id=L611645778>.

[S-86] Daud A, Blank CU, Robert C, Puzanov I, Richtig E, Margolin KA, et al. KEYNOTE-006 study of pembrolizumab (pembro) versus ipilimumab (ipi) for advanced melanoma: efficacy by PD-L1 expression and line of therapy. J Clin Oncol. 2016 May 20;34(15). doi: 10.1200/JCO.2016.34.15_suppl.9513. <http://www.embase.com/search/results?subaction=viewrecord&from=export&id=L611753983>.

[S-87] Mangana J, Cheng PF, Schindler K, Weide B, Held U, Frauchiger AL, et al. Analysis of BRAF and NRAS Mutation Status in Advanced Melanoma Patients Treated with Anti-CTLA-4 Antibodies: Association with Overall Survival? PLoS One. 2015;10(10):e0139438. doi: 10.1371/journal.pone.0139438. <https://www.ncbi.nlm.nih.gov/pubmed/26426340>.

[S-88] Ribas A, Kefford R, Marshall MA, Punt CJA, Haanen JB, Marmol M, et al. Phase III Randomized Clinical Trial Comparing Tremelimumab With Standard-of-Care Chemotherapy in Patients With Advanced Melanoma. J Clin Oncol. 2013 01/07;31(5):616-22. doi: 10.1200/JCO.2012.44.6112. <http://www.ncbi.nlm.nih.gov/pmc/articles/PMC4878048/>.

[S-89] Robert C, Thomas L, Bondarenko I, O'Day S, Weber J, Garbe C, et al. Ipilimumab plus Dacarbazine for Previously Untreated Metastatic Melanoma. N Engl J Med. 2011 Jun 30;364(26):2517-26. doi: 10.1056/NEJMoa1104621. <https://www.nejm.org/doi/full/10.1056/NEJMoa1104621>.

[S-90] Weber JS, Kudchadkar RR, Yu B, Gallenstein D, Horak CE, Inzunza HD, et al. Safety, efficacy, and biomarkers of nivolumab with vaccine in ipilimumab-refractory or -naive melanoma. J Clin Oncol. 2013 Dec 01;31(34):4311-8. doi: 10.1200/jco.2013.51.4802. <https://www.ncbi.nlm.nih.gov/pubmed/24145345>.

[S-91] Algazi AP, Tsai KK, Shoushtari AN, Munhoz RR, Eroglu Z, Piulats JM, et al. Clinical outcomes in metastatic uveal melanoma treated with PD-1 and PD-L1 antibodies. Cancer. 2016 Nov 15;122(21):3344-53. doi: 10.1002/cncr.30258. <https://www.ncbi.nlm.nih.gov/pubmed/27533448>.

[S-92] Arenberger P, Fialova A, Gkalpakiotis S, Pavlikova A, Puzanov I, Arenbergerova M. Melanoma antigens are biomarkers for ipilimumab response. J Eur Acad Dermatol Venereol. 2017 Feb;31(2):252-9. doi: 10.1111/jdv.13940. <https://www.ncbi.nlm.nih.gov/pubmed/27557295>.

[S-93] Chakravarti N, Ivan D, Trinh VA, Glitza IC, Curry JL, Torres-Cabala C, et al. High cytotoxic T-lymphocyte-associated antigen 4 and phospho-AKT expression in tumor samples predicts poor clinical outcomes in ipilimumab-treated melanoma patients. Melanoma Res. 2017 Feb;27(1):24-31. doi: 10.1097/cmr.0000000000000305. <https://www.ncbi.nlm.nih.gov/pubmed/27768639>.

[S-94] Dick J, Lang N, Slynko A, Kopp-Schneider A, Schulz C, Dimitrakopoulou-Strauss A, et al. Use of LDH and autoimmune side effects to predict response to ipilimumab treatment. Immunotherapy. 2016 Sep;8(9):1033-44. doi: 10.2217/imt-2016-0083. <https://www.ncbi.nlm.nih.gov/pubmed/27485076>.

[S-95] Diem S, Kasenda B, Spain L, Martin-Liberal J, Marconcini R, Gore M, et al. Serum lactate dehydrogenase as an early marker for outcome in patients treated with anti-PD-1 therapy in metastatic melanoma. Br J Cancer. 2016 Feb 02;114(3):256-61. doi: 10.1038/bjc.2015.467. <https://www.ncbi.nlm.nih.gov/pubmed/26794281>.

[S-96] Felix J, Cassinat B, Porcher R, Schlageter MH, Maubec E, Pages C, et al. Relevance of serum biomarkers associated with melanoma during follow-up of anti-CTLA-4 immunotherapy. Int Immunopharmacol. 2016 Nov;40:466-73. doi: 10.1016/j.intimp.2016.09.030. <https://www.ncbi.nlm.nih.gov/pubmed/27728898>.

[S-97] Heppt MV, Heinzerling L, Kahler KC, Forschner A, Kirchberger MC, Loquai C, et al. Prognostic factors and outcomes in metastatic uveal melanoma treated with programmed cell death-1 or combined PD-1/cytotoxic T-lymphocyte antigen-4 inhibition. Eur J Cancer. 2017 Jun 21;82:56-65. doi: 10.1016/j.ejca.2017.05.038. <https://www.ncbi.nlm.nih.gov/pubmed/28648699>.

[S-98] Johnson DB, Lovly CM, Flavin M, Panageas KS, Ayers GD, Zhao Z, et al. Impact of NRAS mutations for patients with advanced melanoma treated with immune therapies. Cancer Immunol Res. 2015 Mar;3(3):288-95. doi: 10.1158/2326-6066.cir-14-0207. <http://www.embase.com/search/results?subaction=viewrecord&from=export&id=L609230148http://dx.doi.org/10.1158/2326-6066.CIR-14-0207>.

[S-99] Johnson DB, Frampton GM, Rioth MJ, Yusko E, Xu Y, Guo X, et al. Targeted next generation sequencing identifies markers of response to PD-1 blockade. Cancer Immunol Res. 2016 Nov;4(11):959-67. doi: 10.1158/2326-6066.cir-16-0143. <http://www.embase.com/search/results?subaction=viewrecord&from=export&id=L614953215http://dx.doi.org/10.1158/2326-6066.CIR-16-0143>.

[S-100] Ku GY, Yuan J, Page DB, Schroeder SE, Panageas KS, Carvajal RD, et al. Single-institution experience with ipilimumab in advanced melanoma patients in the compassionate use setting: lymphocyte count after 2 doses correlates with survival. Cancer. 2010 Apr 01;116(7):1767-75. doi: 10.1002/cncr.24951. <https://www.ncbi.nlm.nih.gov/pubmed/20143434>.

[S-101] Larkin J, Lao CD, Urba WJ, McDermott DF, Horak C, Jiang J, et al. Efficacy and safety of nivolumab in patients with BRAF V600 mutant and BRAF wild-type advanced melanoma: a pooled analysis of 4 clinical trials. JAMA Oncol. 2015 Jul;1(4):433-40. doi: 10.1001/jamaoncol.2015.1184. <https://www.ncbi.nlm.nih.gov/pubmed/26181250>.

[S-102] Martens A, Wistuba-Hamprecht K, Geukes Foppen M, Yuan J, Postow MA, Wong P, et al. Baseline Peripheral Blood Biomarkers Associated with Clinical Outcome of Advanced Melanoma Patients Treated with Ipilimumab. Clin Cancer Res. 2016 Jun 15;22(12):2908-18. doi: 10.1158/1078-0432.ccr-15-2412. <https://www.ncbi.nlm.nih.gov/pubmed/26787752>.

[S-103] Morrison C, Pabla S, Nesline M, Gandhi S, Pandey M, Conroy J, et al. The inflamed phenotype in PD-L1 negative melanoma and response to checkpoint inhibitors. J Immunol. 2017;198(1). <http://www.embase.com/search/results?subaction=viewrecord&from=export&id=L617354876>.

[S-104] Roh W, Chen PL, Reuben A, Spencer CN, Prieto PA, Miller JP, et al. Integrated molecular analysis of tumor biopsies on sequential CTLA-4 and PD-1 blockade reveals markers of response and resistance. Sci Transl Med. 2017 Mar 1;9(379). doi: 10.1126/scitranslmed.aah3560. <http://www.embase.com/search/results?subaction=viewrecord&from=export&id=L614722297http://dx.doi.org/10.1126/scitranslmed.aah3560>.

[S-105] Roszik J, Haydu LE, Hess KR, Oba J, Joon AY, Siroy AE, et al. Novel algorithmic approach predicts tumor mutation load and correlates with immunotherapy clinical outcomes using a defined gene mutation set. BMC Med. 2016 Oct 25;14(1):168. doi: 10.1186/s12916-016-0705-4. <https://www.ncbi.nlm.nih.gov/pubmed/27776519>.

[S-106] Sade-Feldman M, Kanterman J, Klieger Y, Ish-Shalom E, Olga M, Saragovi A, et al. Clinical significance of circulating CD33+ CD11bHLA-DR myeloid cells in patients with stage IV melanoma treated with ipilimumab. Clin Cancer Res. 2016 Dec 1;22(23):5661-72. doi: 10.1158/1078-0432.ccr-15-3104. <http://www.embase.com/search/results?subaction=viewrecord&from=export&id=L613703882http://dx.doi.org/10.1158/1078-0432.CCR-15-3104>.

[S-107] Saenger Y, Magidson J, Liaw B, de Moll E, Harcharik S, Fu Y, et al. Blood mRNA expression profiling predicts survival in patients treated with tremelimumab. Clin Cancer Res. 2014 Jun 15;20(12):3310-8. doi: 10.1158/1078-0432.ccr-13-2906. <https://www.ncbi.nlm.nih.gov/pubmed/24721645>.

[S-108] Sim B, Elsheikh S. Expression of programmed death-1 in cutaneous malignant melanoma and its prognostic significance. Br J Dermatol. 2016 Jul;175:133. doi: 10.1111/bjd.14574. <http://www.embase.com/search/results?subaction=viewrecord&from=export&id=L613932312http://dx.doi.org/10.1111/bjd.14574>.

[S-109] Wilgenhof S, Du Four S, Vandenbroucke F, Everaert H, Salmon I, Lienard D, et al. Single-center experience with ipilimumab in an expanded access program for patients with pretreated advanced melanoma. J Immunother. 2013 Apr;36(3):215-22. doi: 10.1097/CJI.0b013e31828eed39. <https://www.ncbi.nlm.nih.gov/pubmed/23502769>.

[S-110] Wistuba-Hamprecht K, Martens A, Heubach F, Romano E, Geukes Foppen M, Yuan J, et al. Peripheral CD8 effector-memory type 1 T-cells correlate with outcome in ipilimumab-treated stage IV melanoma patients. Eur J Cancer. 2017 Mar;73:61-70. doi: 10.1016/j.ejca.2016.12.011. <https://www.ncbi.nlm.nih.gov/pubmed/28167454>.

[S-111] Wu X, Giobbie-Hurder A, Liao X, Connelly C, Connolly EM, Li J, et al. Angiopoietin-2 as a biomarker and target for immune checkpoint therapy. Cancer Immunol Res. 2017 Jan;5(1):17-28. doi: 10.1158/2326-6066.cir-16-0206. <https://www.ncbi.nlm.nih.gov/pubmed/28003187>.

[S-112] Yuan J, Zhou J, Dong Z, Tandon S, Kuk D, Panageas KS, et al. Pretreatment serum VEGF is associated with clinical response and overall survival in advanced melanoma patients treated with ipilimumab. Cancer Immunol Res. 2014 Feb;2(2):127-32. doi: 10.1158/2326-6066.cir-13-0163. <https://www.ncbi.nlm.nih.gov/pubmed/24778276>.

[S-113] Zhou J, Mahoney KM, Giobbie-Hurder A, Zhao F, Lee S, Liao X, et al. Soluble PD-L1 as a biomarker in malignant melanoma treated with checkpoint blockade. Cancer Immunol Res. 2017 Jun;5(6):480-92. doi: 10.1158/2326-6066.cir-16-0329. <http://www.embase.com/search/results?subaction=viewrecord&from=export&id=L616721565http://dx.doi.org/10.1158/2326-6066.CIR-16-0329>.

[S-114] Chasseuil E, Saint-Jean M, Chasseuil H, Peuvrel L, Quéreux G, Nguyen JM, et al. Blood predictive biomarkers for nivolumab in advanced melanoma. Acta Dermato-Venereologica. 2018 Apr 16;98(4):406-10. doi: 10.2340/00015555-2872. <http://www.embase.com/search/results?subaction=viewrecord&from=export&id=L621674753> <http://dx.doi.org/10.2340/00015555-2872>.

[S-115] Gaudy-Marqueste C, Dussouil AS, Carron R, Troin L, Malissen N, Loundou A, et al. Survival of melanoma patients treated with targeted therapy and immunotherapy after systematic upfront control of brain metastases by radiosurgery. Eur J Cancer. 2017 Oct;84:44-54. doi: 10.1016/j.ejca.2017.07.017. <https://www.sciencedirect.com/science/article/pii/S0959804917311279?via%3Dihub>.

[S-116] Kaufman HL, Russell J, Hamid O, Bhatia S, Terheyden P, D'Angelo SP, et al. Avelumab in patients with chemotherapy-refractory metastatic Merkel cell carcinoma: a multicentre, single-group, open-label, phase 2 trial. Lancet Oncol. 2016 Oct;17(10):1374-85. doi: 10.1016/s1470-2045(16)30364-3. <https://www.ncbi.nlm.nih.gov/pubmed/27592805>.

[S-117] Shapiro I, Grote HJ, D'Urso V, Von Heydebreck A, Mahnke L, Kaufman H, et al. Exploratory biomarker analysis in avelumab-treated patients with metastatic Merkel cell carcinoma progressed after chemotherapy. J Clin Oncol. 2017 May 20;35(15). doi: 10.1200/JCO.2017.35.15_suppl.9557. <http://www.embase.com/search/results?subaction=viewrecord&from=export&id=L617435529>.

[S-118] Motzer RJ, Escudier B, McDermott DF, George S, Hammers HJ, Srinivas S, et al. Nivolumab versus Everolimus in Advanced Renal-Cell Carcinoma. N Engl J Med. 2015 Nov 5;373(19):1803-13. doi: 10.1056/NEJMoa1510665. <https://www.nejm.org/doi/full/10.1056/NEJMoa1510665>.

[S-119] Atkins MB, McDermott DF, Powles T, Motzer RJ, Rini BI, Fong L, et al. IMmotion150: A phase II trial in untreated metastatic renal cell carcinoma (mRCC) patients (pts) of atezolizumab (atezo) and bevacizumab (bev) vs and following atezo or sunitinib (sun). J Clin Oncol. 2017;35(15). <http://www.embase.com/search/results?subaction=viewrecord&from=export&id=L617388476>.

[S-120] Grünwald V, McDermott DF, Atkins M, Motzer R, Rini B, Escudier B, et al. A phase II study of atezolizumab (atezo) with or without bevacizumab (bev) vs sunitinib (sun) in untreated metastatic renal cell carcinoma (mRCC) patients (pts). Oncol Res Treat. 2017;40:113-4. doi: 10.1159/000479566. <http://www.embase.com/search/results?subaction=viewrecord&from=export&id=L618608438> <http://dx.doi.org/10.1159/000479566>

<https://www.karger.com/Article/Pdf/479566>.

[S-121] McDermott DF, Sosman JA, Sznol M, Massard C, Gordon MS, Hamid O, et al. Atezolizumab, an Anti-Programmed Death-Ligand 1 Antibody, in Metastatic Renal Cell Carcinoma: Long-Term Safety, Clinical Activity, and Immune Correlates From a Phase Ia Study. J Clin Oncol. 2016 Mar 10;34(8):833-42. doi: 10.1200/jco.2015.63.7421. <https://www.ncbi.nlm.nih.gov/pubmed/26755520>.

[S-122] Motzer RJ, Tannir NM, McDermott DF, Frontera OA, Melichar B, Plimack ER, et al. Nivolumab + Ipilimumab (N+I) vs Sunitinib (S) for treatment-naïve advanced or metastatic renal cell carcinoma (aRCC): Results from CheckMate 214, including overall survival by subgroups. J Immunother Cancer. 2017;5(S3). doi: 10.1186/s40425-017-0297-3. <http://www.embase.com/search/results?subaction=viewrecord&from=export&id=L620120794> <http://dx.doi.org/10.1186/s40425-017-0297-3>

<https://jitc.biomedcentral.com/track/pdf/10.1186/s40425-017-0297-3>.

[S-123] Boku N, Kang Y, Satoh T, Chao Y, Kato K, Chung H, et al. A Phase 3 Study of nivolumab (Nivo) in previously treated advanced gastric or gastroesophageal junction (G/GEJ) cancer: Updated results and subset analysis by PD-L1 expression (ATTRACTION-02). Ann Oncol. 2017 Sep;28(Suppl 5):209. doi: 10.1093/annonc/mdx369. <Go to ISI>://WOS:000411324001132.

[S-124] Fuchs CS, Doi T, Jang RWJ, Muro K, Satoh T, Machado M, et al. KEYNOTE-059 cohort 1: Efficacy and safety of pembrolizumab (pembro) monotherapy in patients with previously treated advanced gastric cancer. J Clin Oncol. 2017 May 20;35(15). doi: 10.1200/JCO.2017.35.15_suppl.4003. <http://www.embase.com/search/results?subaction=viewrecord&from=export&id=L617388480>.

[S-125] Andre T, Overman M, Lonardi S, Aglietta M, McDermott R, Wong K, et al. Analysis of tumor PD-L1 expression and biomarkers in relation to clinical activity in patients (pts) with deficient DNA mismatch repair (dMMR)/high microsatellite instability (MSI-H) metastatic colorectal cancer (mCRC) treated with nivolumab (NIVO) 1 ipilimumab (IPI): CheckMate 142. Ann Oncol. 2017;28(Supplement 5):163.

[S-126] Overman MJ, Lonardi S, Leone F, McDermott RS, Morse MA, Wong KYM, et al. Nivolumab in patients with DNA mismatch repair defficient/microsatellite instability high metastatic colorectal cancer: Update from checkmate 142. J Clin Oncol. 2017;35(4). <http://www.embase.com/search/results?subaction=viewrecord&from=export&id=L618087441>.

[S-127] Diaz LA, Marabelle A, Delord JP, Shapira-Frommer R, Geva R, Peled N, et al. Pembrolizumab therapy for microsatellite instability high (MSI-H) colorectal cancer (CRC) and non-CRC. J Clin Oncol. 2017 May 20;35(15). doi: 10.1200/JCO.2017.35.15_suppl.3071. <http://www.embase.com/search/results?subaction=viewrecord&from=export&id=L617435500>.

[S-128] Diaz L, Marabelle A, Kim TW, Geva R, Van Cutsem E, André T, et al. Efficacy of pembrolizumab in phase 2 KEYNOTE-164 and KEYNOTE-158 studies of microsatellite instability high cancers. Annals of Oncology. 2017 Sep;28:v128-v9. doi: 10.1093/annonc/mdx367.020. <http://www.embase.com/search/results?subaction=viewrecord&from=export&id=L619623337> <http://dx.doi.org/10.1093/annonc/mdx367.020>

<https://watermark.silverchair.com/mdx367.020.pdf?token=AQECAHi208BE49Ooan9kkhW_Ercy7Dm3ZL_9Cf3qfKAc485ysgAAAdEwggHNBgkqhkiG9w0BBwagggG-MIIBugIBADCCAbMGCSqGSIb3DQEHATAeBglghkgBZQMEAS4wEQQM-WXs8XhPhRUPtcUYAgEQgIIBhBfnUUIWlSDete-hFUd-Qcywc5Gn86kJTsrB-faO17U7GPwZ7gZhrLRiac7msu2-minfUrBGhCGw4cXkYhzT_ade7TRD6HIKJEr1wWTkHOz39Xz9i95pXJVm_UDrsz-8Kr_ASvRDdX1Pyee2nbhJMCwrIgsYZFUNSj7pPRCMbIOXemxc1WS0hvh0RJ3LgV74R8HroyPeonIkF50T-kEojTyz5jNzeR3fb74lN3YDpcAtHVIhoUAtFSqJ6GeCvt2fyzOm2lIyHmFY-gVDeCASXQH8Xp5aLOP50rXBzw83KFyA55kpF261E62Wor0iUnnsRFYXqfkDjuQEV_FlwaGzxXF1j60B-S2NwWs9YJzXaq92KFJew47zlxWFvwZOk7Ygb8R1uUjvlUd37gZkuix0HH-E9lNNNM8AqQr5w5hDkvdRqbB6trUGTNaDrLdRkeUWE01l5qiOTZPR7a6p0RlQ2gwRAnHHO_uUv8uTlGAiM6vVx3uKe_6HWIZlS6jnVxCxQcGcsnc>.

[S-129] Jaeger D, Sharma P, Bono P, Kim J, Spiliopoulou P, Calvo E, et al. Nivolumab monotherapy in metastatic urothelial cancer (mUC): Efficacy (by PD-L1 expression) and safety results from the CheckMate 032 study. Oncol Res Treat. 2016 Oct;39:269. doi: 10.1159/000449050. <http://www.embase.com/search/results?subaction=viewrecord&from=export&id=L613153328http://dx.doi.org/10.1159/000449050>.

[S-130] Rosenberg JE, Bono P, Kim J, Spiliopoulou P, Calvo E, Pillai R, et al. Nivolumab monotherapy in metastatic urothelial cancer (mUC): Updated efficacy by subgroups and safety results from the CheckMate 032 study. Ann Oncol. 2016 Oct 1;27. doi: 10.1093/annonc/mdw373.12. <http://www.embase.com/search/results?subaction=viewrecord&from=export&id=L613912051http://dx.doi.org/10.1093/annonc/mdw373.12>.

[S-131] Sharma P, Retz M, Siefker-Radtke A, Baron A, Necchi A, Bedke J, et al. Nivolumab in metastatic urothelial carcinoma after platinum therapy (CheckMate 275): a multicentre, single-arm, phase 2 trial. Lancet Oncol. 2017 Mar;18(3):312-22. doi: 10.1016/s1470-2045(17)30065-7. <https://www.ncbi.nlm.nih.gov/pubmed/28131785>.

[S-132] Galsky MD, Retz M, Siefker-Radtke AO, Baron A, Necchi A, Bedke J, et al. PR Efficacy and safety of nivolumab monotherapy in patients with metastatic urothelial cancer (mUC) who have received prior treatment: Results from the phase II CheckMate 275 study. Ann Oncol. 2016;27(suppl_6). doi: 10.1093/annonc/mdw435.24. <http://www.embase.com/search/results?subaction=viewrecord&from=export&id=L613911561http://dx.doi.org/10.1093/annonc/mdw435.24>.

[S-133] Massard C, Gordon MS, Sharma S, Rafii S, Wainberg ZA, Luke JJ, et al. Safety and efficacy of durvalumab (MEDI4736), a PD-L1 antibody, in urothelial bladder cancer. J Clin Oncol. 2016 May 20;34(15). doi: 10.1200/JCO.2016.34.15_suppl.4502. <http://www.embase.com/search/results?subaction=viewrecord&from=export&id=L611752156>.

[S-134] Bais C, Kuziora M, Morehouse C, Higgs BW, Raja R, Lee Y, et al. Biologic and clinical relevance of an IFNG mRNA signature (IFNGS) and PD-L1 protein expression in tumor and immune cells in urothelial cancer (UC) patients (pts) treated with durvalumab (D). J Clin Oncol. 2017 May 20;35(15). doi: 10.1200/JCO.2017.35.15_suppl.3037. <http://www.embase.com/search/results?subaction=viewrecord&from=export&id=L617435157>.

[S-135] Hahn NM, Powles T, Massard C, Arkenau HT, Friedlander TW, Hoimes CJ, et al. Updated efficacy and tolerability of durvalumab in locally advanced or metastatic urothelial carcinoma (UC). J Clin Oncol. 2017;35(15). <http://www.embase.com/search/results?subaction=viewrecord&from=export&id=L617435486>.

[S-136] Powles T, Durán I, van der Heijden MS, Loriot Y, Vogelzang NJ, De Giorgi U, et al. Atezolizumab versus chemotherapy in patients with platinum-treated locally advanced or metastatic urothelial carcinoma (IMvigor211): A multicentre, open-label, phase 3 randomised controlled trial. Lancet. 2018 Feb 24;391(10122):748-57. doi: 10.1016/S0140-6736(17)33297-X. <http://www.embase.com/search/results?subaction=viewrecord&from=export&id=L619909227> <http://dx.doi.org/10.1016/S0140-6736(17)33297-X>

<https://www.sciencedirect.com/science/article/pii/S014067361733297X?via%3Dihub>.

[S-137] Balar AV, Galsky MD, Rosenberg JE, Powles T, Petrylak DP, Bellmunt J, et al. Atezolizumab as first-line treatment in cisplatin-ineligible patients with locally advanced and metastatic urothelial carcinoma: a single-arm, multicentre, phase 2 trial. Lancet. 2017 Jan 07;389(10064):67-76. doi: 10.1016/s0140-6736(16)32455-2. <https://www.ncbi.nlm.nih.gov/pubmed/27939400>.

[S-138] Rosenberg JE, Hoffman-Censits J, Powles T, van der Heijden MS, Balar AV, Necchi A, et al. Atezolizumab in patients with locally advanced and metastatic urothelial carcinoma who have progressed following treatment with platinum-based chemotherapy: a single-arm, multicentre, phase 2 trial. Lancet. 2016 May 07;387(10031):1909-20. doi: 10.1016/s0140-6736(16)00561-4. <https://www.ncbi.nlm.nih.gov/pubmed/26952546>.

[S-139] Rosenberg JE, Petrylak DP, Van Der Heijden MS, Necchi A, O'Donnell PH, Loriot Y, et al. PD-L1 expression, cancer genome atlas (TCGA) subtype, and mutational load as independent predictors of response to atezolizumab (ATEZO) in metastatic urothelial carcinoma (mUC; IMvigor210). J Clin Oncol. 2016;34. <http://www.embase.com/search/results?subaction=viewrecord&from=export&id=L611752035>.

[S-140] Dirix LY, Takacs I, Jerusalem G, Nikolinakos P, Arkenau HT, Forero-Torres A, et al. Avelumab, an anti-PD-L1 antibody, in patients with locally advanced or metastatic breast cancer: a phase 1b JAVELIN Solid Tumor study. Breast Cancer Res Treat. 2017 Feb;167(3):1-16. doi: 10.1007/s10549-017-4537-5. <http://www.embase.com/search/results?subaction=viewrecord&from=export&id=L618923675> <http://dx.doi.org/10.1007/s10549-017-4537-5>

<https://link.springer.com/content/pdf/10.1007%2Fs10549-017-4537-5.pdf>.

[S-141] Bellmunt J, de Wit R, Vaughn DJ, Fradet Y, Lee JL, Fong L, et al. Pembrolizumab as Second-Line Therapy for Advanced Urothelial Carcinoma. N Engl J Med. 2017 Mar 16;376(11):1015-26. doi: 10.1056/NEJMoa1613683. <https://www.ncbi.nlm.nih.gov/pubmed/28212060>.

[S-142] Bajorin DF, De Wit R, Vaughn DJ, Fradet Y, Lee JL, Fong L, et al. Planned survival analysis from KEYNOTE-045: Phase 3, open-label study of pembrolizumab (pembro) versus paclitaxel, docetaxel, or vinflunine in recurrent, advanced urothelial cancer (UC). J Clin Oncol. 2017;35(15). <http://www.embase.com/search/results?subaction=viewrecord&from=export&id=L617388509>.

[S-143] O'Donnell PH, Grivas P, Balar AV, Bellmunt J, Vuky J, Powles T, et al. Biomarker findings and mature clinical results from KEYNOTE-052: First-line pembrolizumab (pembro) in cisplatin-ineligible advanced urothelial cancer (UC). J Clin Oncol. 2017 May 20;35(15). doi: 10.1200/JCO.2017.35.15_suppl.4502. <http://www.embase.com/search/results?subaction=viewrecord&from=export&id=L617388416>.

[S-144] Balar A, Bellmunt J, O'Donnell PH, Castellano D, Grivas P, Vuky J, et al. PR Pembrolizumab (pembro) as first-line therapy for advanced/unresectable or metastatic urothelial cancer: Preliminary results from the phase 2 KEYNOTE-052 study. Ann Oncol. 2016 Oct 1;27. doi: 10.1093/annonc/mdw435.25. <http://www.embase.com/search/results?subaction=viewrecord&from=export&id=L613912244http://dx.doi.org/10.1093/annonc/mdw435.25>.

[S-145] Petrylak DP, Powles T, Bellmunt J, Braiteh FS, Loriot Y, Zambrano CC, et al. A phase Ia study of MPDL3280A (anti-PDL1): Updated response and survival data in urothelial bladder cancer (UBC). J Clin Oncol. 2015 May 20;33(15). doi: DOI 10.1200/jco.2015.33.15_suppl.4501. <http://www.embase.com/search/results?subaction=viewrecord&from=export&id=L72013251>.

[S-146] Teo MY, Seier K, Ostrovnaya I, Regazzi AM, Kania BE, Moran MM, et al. Alterations in DNA Damage Response and Repair Genes as Potential Marker of Clinical Benefit From PD-1/PD-L1 Blockade in Advanced Urothelial Cancers. J Clin Oncol. 2018 Feb 28;36(17):Jco2017757740. doi: 10.1200/jco.2017.75.7740. <https://www.ncbi.nlm.nih.gov/pubmed/29489427>.

[S-147] Ferris RL, Blumenschein G, Jr., Fayette J, Guigay J, Colevas AD, Licitra L, et al. Nivolumab for Recurrent Squamous-Cell Carcinoma of the Head and Neck. N Engl J Med. 2016 Nov 10;375(19):1856-67. doi: 10.1056/NEJMoa1602252. <https://www.ncbi.nlm.nih.gov/pubmed/27718784>.

[S-148] Ferris RL, Licitra L, Fayette J, Even C, Blumenschein GR, Harrington K, et al. Nivolumab (Nivo) vs investigator's choice (IC) in patients with recurrent or metastatic (R/M) squamous cell carcinoma of the head and neck (SCCHN): Efficacy and safety in CheckMate 141 by prior cetuximab use. J Clin Oncol. 2017;35(15). <http://www.embase.com/search/results?subaction=viewrecord&from=export&id=L617389010>.

[S-149] Ferris RL, Blumenschein GR, Fayette J, Guigay J, Colevas AD, Licitra LF, et al. Further evaluations of nivolumab (nivo) versus investigator's choice (IC) chemotherapy for recurrent or metastatic (R/M) squamous cell carcinoma of the head and neck (SCCHN): CheckMate 141. J Clin Oncol. 2016;34. <http://www.embase.com/search/results?subaction=viewrecord&from=export&id=L611752189>.

[S-150] Ferris RL, Blumenschein G, Fayette J, Guigay J, Colevas AD, Licitra L, et al. Nivolumab vs investigator's choice in recurrent or metastatic squamous cell carcinoma of the head and neck: 2-year long-term survival update of CheckMate 141 with analyses by tumor PD-L1 expression. Oral Oncol. 2018 Jun;81:45-51. doi: 10.1016/j.oraloncology.2018.04.008. <http://www.embase.com/search/results?subaction=viewrecord&from=export&id=L2000663176> <http://dx.doi.org/10.1016/j.oraloncology.2018.04.008> <https://www.sciencedirect.com/science/article/pii/S1368837518301490?via%3Dihub>.

[S-151] Chow LQ, Haddad R, Gupta S, Mahipal A, Mehra R, Tahara M, et al. Antitumor Activity of Pembrolizumab in Biomarker-Unselected Patients With Recurrent and/or Metastatic Head and Neck Squamous Cell Carcinoma: Results From the Phase Ib KEYNOTE-012 Expansion Cohort. J Clin Oncol. 2016 Sep 19;34(32):3838-45. doi: 10.1200/jco.2016.68.1478. <https://www.ncbi.nlm.nih.gov/pubmed/27646946>.

[S-152] Chow LQM, Mehra R, Haddad RI, Mahipal A, Weiss J, Berger R, et al. Biomarkers and response to pembrolizumab (pembro) in recurrent/metastatic head and neck squamous cell carcinoma (R/M HNSCC). J Clin Oncol. 2016 May 20;34(15). doi: 10.1200/JCO.2016.34.15_suppl.6010. <http://www.embase.com/search/results?subaction=viewrecord&from=export&id=L611752258>.

[S-153] Younes A, Santoro A, Shipp M, Zinzani PL, Timmerman JM, Ansell S, et al. Nivolumab for classical Hodgkin's lymphoma after failure of both autologous stem-cell transplantation and brentuximab vedotin: a multicentre, multicohort, single-arm phase 2 trial. Lancet Oncol. 2016 Sep;17(9):1283-94. doi: 10.1016/s1470-2045(16)30167-x. <https://www.ncbi.nlm.nih.gov/pubmed/27451390>.

[S-154] Roemer MGM, Ligon AH, Engert A, Younes A, Santoro A, Zinzani PL, et al. Chromosome 9p24.1/PD-L1/PD-L2-alterations and PD-L1 expression and treatment outcomes in patients with classical hodgkin lymphoma treated with nivolumab (PD-1 blockade). Blood. 2016;128(22). <http://www.embase.com/search/results?subaction=viewrecord&from=export&id=L614247596>.

[S-155] De Remigis A, de Gruijl TD, Uram JN, Tzou SC, Iwama S, Talor MV, et al. Development of thyroglobulin antibodies after GVAX immunotherapy is associated with prolonged survival. Int J Cancer. 2015 Jan 01;136(1):127-37. doi: 10.1002/ijc.28973. <https://www.ncbi.nlm.nih.gov/pubmed/24832153>.

[S-156] Adams S, Schmid P, Rugo HS, Winer EP, Loirat D, Awada A, et al. Phase 2 study of pembrolizumab (pembro) monotherapy for previously treated metastatic triple-negative breast cancer (mTNBC): KEYNOTE-086 cohort A. J Clin Oncol. 2017 May 20;35(15). doi: 10.1200/JCO.2017.35.15_suppl.1008. <http://www.embase.com/search/results?subaction=viewrecord&from=export&id=L617388684>.

[S-157] Ayers M, Levitan D, Arreaza G, Liu F, Mogg R, Bang YJ, et al. Association between microsatellite instability and clinical response across tumor types in the phase Ib KEYNOTE-012 and KEYNOTE-028 studies of pembrolizumab in PD-L1-expressing advanced solid tumors. J Immunother Cancer. 2016;4(S1). doi: 10.1186/s40425-016-0172-7. <http://www.embase.com/search/results?subaction=viewrecord&from=export&id=L613518866http://dx.doi.org/10.1186/s40425-016-0172-7>.

[S-158] Navarro A, Arance A, Reguart N, Paré L, Galván P, Marti AM, et al. Association of response to programmed death 1 receptor or ligand (PD1/PDL1) blockade with immune-related gene expression profiling across three cancer-types. J Clin Oncol. 2016 May 20;34(15). doi: 10.1200/JCO.2016.34.15_suppl.3038. <http://www.embase.com/search/results?subaction=viewrecord&from=export&id=L611752031>.

[S-159] Prat A, Navarro A, Pare L, Reguart N, Galvan P, Pascual T, et al. Immune-Related Gene Expression Profiling After PD-1 Blockade in Non-Small Cell Lung Carcinoma, Head and Neck Squamous Cell Carcinoma, and Melanoma. Cancer Res. 2017 Jul 01;77(13):3540-50. doi: 10.1158/0008-5472.can-16-3556. <https://www.ncbi.nlm.nih.gov/pubmed/28487385>.

[S-160] Legrand F. Association of high tissue TMB and atezolizumab efficacy across multiple tumor types. The American Society of Clinical Oncology. Chicago, Illinois; 2018.
